# Deciphering the RNA landscapes on mammalian cell surfaces

**DOI:** 10.1093/procel/pwaf079

**Published:** 2025-09-18

**Authors:** Xiao Jiang, Chu Xu, Enzhuo Yang, Danhua Xu, Yong Peng, Xue Han, Jingwen Si, Qixin Shao, Zhuo Liu, Qiuxiao Chen, Weizhi He, Shuang He, Yanhui Xu, Chuan He, Xinxin Huang, Lulu Hu

**Affiliations:** Cancer Institute, Fudan University Shanghai Cancer Center, Shanghai Key Laboratory of Medical Epigenetics, International Laboratory of Medical Epigenetics and Metabolism, Ministry of Science and Technology, Institutes of Biomedical Sciences, Fudan University, Shanghai 200032, China; Cancer Institute, Fudan University Shanghai Cancer Center, Shanghai Key Laboratory of Medical Epigenetics, International Laboratory of Medical Epigenetics and Metabolism, Ministry of Science and Technology, Institutes of Biomedical Sciences, Fudan University, Shanghai 200032, China; Cancer Institute, Fudan University Shanghai Cancer Center, Shanghai Key Laboratory of Medical Epigenetics, International Laboratory of Medical Epigenetics and Metabolism, Ministry of Science and Technology, Institutes of Biomedical Sciences, Fudan University, Shanghai 200032, China; Shanghai Xuhui Central Hospital, Zhongshan-Xuhui Hospital, and the Shanghai Key Laboratory of Medical Epigenetics, the International Co-Laboratory of Medical Epigenetics and Metabolism (Ministry of Science and Technology), Institutes of Biomedical Sciences, Fudan University, Shanghai 200032, China; Innovative Institute of Chinese Medicine and Pharmacy, Chengdu University of Traditional Chinese Medicine, Chengdu 611137, China; Institute of Herbgenomics, Chengdu University of Traditional Chinese Medicine, Chengdu 611137, China; Cancer Institute, Fudan University Shanghai Cancer Center, Shanghai Key Laboratory of Medical Epigenetics, International Laboratory of Medical Epigenetics and Metabolism, Ministry of Science and Technology, Institutes of Biomedical Sciences, Fudan University, Shanghai 200032, China; Sycamore Research Institute of Life Sciences, Shanghai 201203, China; Shanghai Key Laboratory of Signaling and Disease Research, Frontier Science Center for Stem Cell Research, School of Life Sciences and Technology, Tongji University, Shanghai 200092, China; Cancer Institute, Fudan University Shanghai Cancer Center, Shanghai Key Laboratory of Medical Epigenetics, International Laboratory of Medical Epigenetics and Metabolism, Ministry of Science and Technology, Institutes of Biomedical Sciences, Fudan University, Shanghai 200032, China; Cancer Institute, Fudan University Shanghai Cancer Center, Shanghai Key Laboratory of Medical Epigenetics, International Laboratory of Medical Epigenetics and Metabolism, Ministry of Science and Technology, Institutes of Biomedical Sciences, Fudan University, Shanghai 200032, China; Cancer Institute, Fudan University Shanghai Cancer Center, Shanghai Key Laboratory of Medical Epigenetics, International Laboratory of Medical Epigenetics and Metabolism, Ministry of Science and Technology, Institutes of Biomedical Sciences, Fudan University, Shanghai 200032, China; Cancer Institute, Fudan University Shanghai Cancer Center, Shanghai Key Laboratory of Medical Epigenetics, International Laboratory of Medical Epigenetics and Metabolism, Ministry of Science and Technology, Institutes of Biomedical Sciences, Fudan University, Shanghai 200032, China; Cancer Institute, Fudan University Shanghai Cancer Center, Shanghai Key Laboratory of Medical Epigenetics, International Laboratory of Medical Epigenetics and Metabolism, Ministry of Science and Technology, Institutes of Biomedical Sciences, Fudan University, Shanghai 200032, China; Cancer Institute, Fudan University Shanghai Cancer Center, Shanghai Key Laboratory of Medical Epigenetics, International Laboratory of Medical Epigenetics and Metabolism, Ministry of Science and Technology, Institutes of Biomedical Sciences, Fudan University, Shanghai 200032, China; Department of Chemistry, Institute for Biophysical Dynamics, The University of Chicago, Chicago, IL 60637, United States; Department of Biochemistry and Molecular Biology, Institute for Biophysical Dynamics, The University of Chicago, Chicago, IL 60637, United States; Howard Hughes Medical Institute, The University of Chicago, Chicago, IL 60637, United States; Shanghai Xuhui Central Hospital, Zhongshan-Xuhui Hospital, and the Shanghai Key Laboratory of Medical Epigenetics, the International Co-Laboratory of Medical Epigenetics and Metabolism (Ministry of Science and Technology), Institutes of Biomedical Sciences, Fudan University, Shanghai 200032, China; Cancer Institute, Fudan University Shanghai Cancer Center, Shanghai Key Laboratory of Medical Epigenetics, International Laboratory of Medical Epigenetics and Metabolism, Ministry of Science and Technology, Institutes of Biomedical Sciences, Fudan University, Shanghai 200032, China; Sycamore Research Institute of Life Sciences, Shanghai 201203, China

**Keywords:** surface RNA landscapes, mammalian blood cells, high-throughput sequencing, live cell imaging and quantification

## Abstract

Cell surface RNAs, notably glycoRNAs, have been reported, yet the precise compositions of surface RNAs across different primary cell types remain unclear. Here, we introduce a comprehensive suite of methodologies for profiling, imaging, and quantifying specific surface RNAs. We present AMOUR, a method leveraging T7-based linear amplification, to profile surface RNAs while preserving plasma membrane integrity. By integrating fluorescently labeled DNA probes with live primary cells, and employing imaging along with flow cytometry analysis, we can effectively image and quantify representative surface RNAs. Utilizing these techniques, we have identified diverse non-coding RNAs present on mammalian cell surfaces, expanding beyond the known glycoRNAs. We confirm the membrane anchorage and quantify the abundance of several representative surface RNA molecules in cultured HeLa cells and human umbilical cord blood mononuclear cells (hUCB-MNCs). Our imaging and flow cytometry analyses unequivocally confirm the membrane localization of Y family RNAs, spliceosomal snRNA *U5*, mitochondrial rRNA *MTRNR2*, mitochondrial tRNA *MT-TA*, *VTRNA1-1*, and the long non-coding RNA *XIST*. Our study not only introduces effective approaches for investigating surface RNAs but also provides a detailed portrayal of the surface RNA landscapes of hUCB-MNCs and murine blood cells, paving the way for future research in the field of surface RNAs.

## Introduction

Cellular life thrives within the confinements of the cell membrane, the defining boundary that contains functional units. Traditionally recognized as a 4-nm (Danielli and Harvey, 1935) lipid bilayer (Gorter and Grendel, 1925) encompassing lipids, proteins, and glycans (Cole, 1932; Edidin, 2003; Mayor *et al.*, 2023), the plasma membrane contains mosaic proteins embedded within the fluid lipid bilayer (Maselli *et al.*, 2015; Simons and Ikonen, 1997) and cholesterol-enriched lipid rafts (Brown and Rose, 1992), acting as potential signaling platforms.

The “RNA world” theory (Gilbert, 1986) suggests an RNA-centric pre-cellular era, indicating RNA’s dual capacity to store genetic information and also catalyze reactions as ribozymes (Cech, 1986; Guerrier-Takada and Altman, 1984; Guerrier-Takada *et al.*, 1983; Kruger *et al.*, 1982; Zaug and Cech, 1986). The “RNA world” theory may also suggest that RNA could be encapsulated by membrane bilayer or even be part of the membrane during the evolution of life. Advancements in imaging, sequencing, and mass spectrometry urged a re-evaluation of the plasma membrane’s composition. This prompted an inquiry into whether RNA also associates with the membrane. Indeed, a recent breakthrough discovered the presence of non-coding RNAs and fragments of protein-coding RNAs on mammalian cell surfaces (Huang *et al.*, 2020). Moreover, small RNAs, including tRNAs, have been reported to be N-glycosylated at acp^3^U and displayed on cell surfaces (Flynn *et al.*, 2021; Xie *et al.*, 2024), along with findings showing interactions between short guanine-rich RNAs and artificial unilamellar vesicles *in vitro* (Czerniak and Saenz, 2022). Advances in techniques like spatial imaging assay further offered new views into glycoRNAs’ roles in cell–cell interactions during immune responses (Ma *et al.*, 2024).

Despite recent discoveries indicating the presence of multiple RNAs, notably glycoRNAs, residing on the plasma membrane (Flynn *et al.*, 2021; Huang *et al.*, 2020; Xie *et al.*, 2024), non-disruptive methodologies that enable comprehensive and precise profiling of global surface RNAs, particularly within rare cell populations, remain elusive. Crucial aspects, such as the potential diverse nature of surface RNAs and their interaction with the plasma membrane, necessitate thorough exploration. GlycoRNAs on the cell surface of murine neutrophils have been implicated in neutrophil recruitment to inflammatory sites and subsequent migration through endothelial cells (Zhang *et al.*, 2024). Additionally, RNA-binding proteins and glycoRNAs have been reported to form nanoclusters that facilitate the entry of cell-penetrating peptides (Perr *et al.*, 2025). Although previous studies have examined total surface RNAs, there remains a critical need for effective mapping techniques and a more detailed understanding of specific surface RNA species within primary cells. To address this gap, we conducted an in-depth analysis of surface RNAs from distinct primary cell types, aiming to elucidate their associations with the respective residing cell types.

We report here a set of new strategies tailored for the investigation of surface RNAs located on the outer plasma membrane across diverse cell types. We discovered diversified non-coding RNAs on the surfaces of human and murine blood cells.

## Results

### RNA localization on the outer membrane surface of cultured cells and live hUCB-MNCs

**Figure 1.**
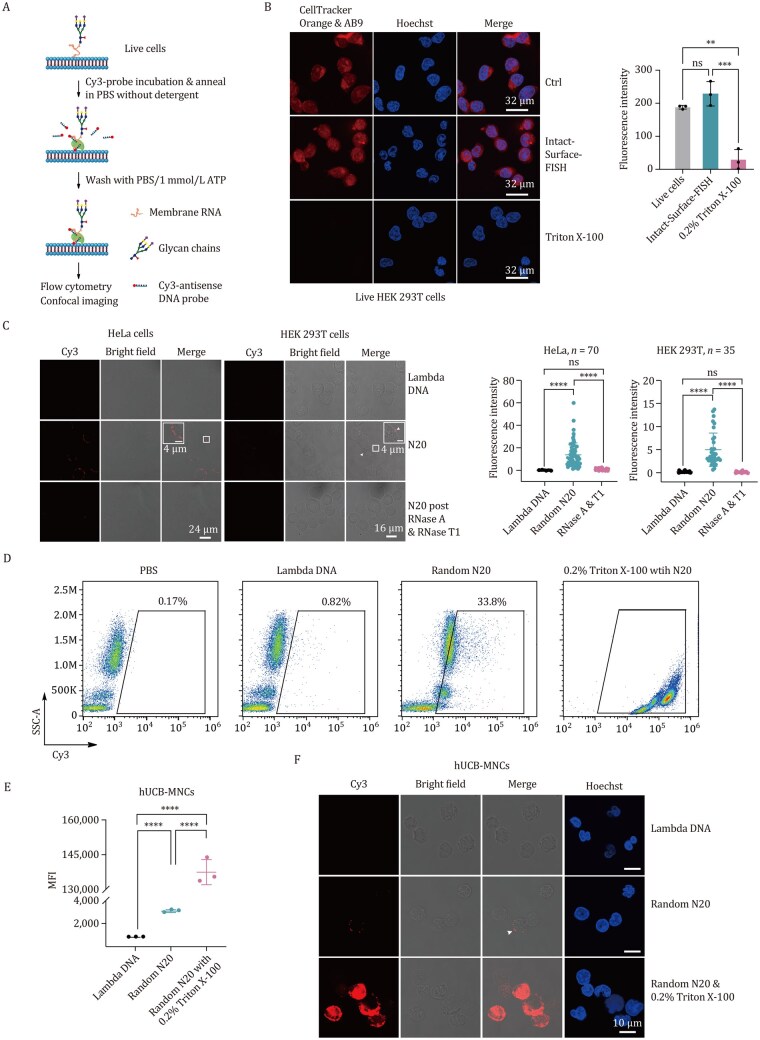
**Localization of RNA molecules on the outer membrane surface of cell lines and primary human cells, identified by Intact-Surface-FISH**. (A) Schematic flow chart of Intact-Surface-FISH. Live primary cells or cell lines are incubated with Cy3-DNA probes complementary to target surface RNAs, washed, and subjected to flow cytometry analysis or confocal imaging. (B) Transmission-through-dye confocal imaging of live HEK 293T cells under various treatments: control (upper panel), Intact-Surface-FISH (middle panel), and 0.2% Triton X-100 treatment prior to Intact-Surface-FISH for 5 min (bottom panel). Cells were stained with the membrane-permeable dye CellTracker Orange and the membrane-impermeant quencher acid blue 9 (AB9). In cells with intact membranes, AB9 is excluded, and CellTracker Orange fluorescence is observed throughout the cell. In cells with compromised membranes, AB9 enters and quenches CellTracker Orange, reducing or eliminating fluorescence signals. Box plots illustrate the mean fluorescence intensity per image for the control group, Intact-Surface-FISH-treated group, and 0.2% Triton X-100 permeabilized group. (C) Confocal imaging of HeLa and HEK 293T cells stained with Cy3-Lambda DNA control and Cy3-N20 DNA probe (*N* represents a random A/T/C/G), without (middle panel) and with (bottom panel) RNase A and T1 pre-treatment, using the Intact-Surface-FISH strategy. Quantitative analysis of mean fluorescence intensity per cell is provided for each treatment. *n* indicates the number of cells examined. (D) Fluorescence labeling of live hUCB-MNCs with Cy3-Lambda DNA control and Cy3-N20 DNA probes complementary to surface RNAs, using Intact-Surface-FISH. The PBS-incubated group serves as a negative control, while 4% Paraformaldehyde (PFA)-fixed cells permeabilized with 0.2% Triton X-100 prior to Cy3-N20 incubation serve as the positive control group. The gated region indicates the cell population displaying specific RNAs on the cell surface. (E) Mean fluorescence intensity (MFI) of live hUCB-MNCs stained with Cy3-Lambda DNA control and Cy3-N20, with or without Triton X-100 permeabilization, identified using Intact-Surface-FISH. (F) Confocal imaging of hUCB-MNCs stained with Cy3-Lambda DNA or Cy3-N20, with or without Triton X-100 permeabilization, using Intact-Surface-FISH. Quantitative analysis of mean fluorescence intensity per cell is provided for each treatment. *n* indicates the number of cells examined. All flow cytometry analyses and confocal imaging data represent three independent experiments with similar results, shown as mean ± SD, and analyzed using an unpaired two-tailed Student’s *t*-test; ns: not significant. **P < 0.01, ***P < 0.001, ****P < 0.0001.

To investigate RNA localization on the outer membranes of mammalian cells, we developed an “Intact-Surface-FISH” assay, enabling surface RNA imaging while preserving cell viability and membrane integrity (Supplementary Methods and Fig. 1A). This method builds upon the Surface-FISH (fluorescence *in situ* hybridization) technique (Huang *et al.*, 2020), optimizing the process by reducing the DNA probe incubation time to 30 min for live primary cells and cultured cell lines. The shortened incubation time is optimized to minimize endocytosis in live primary cells and cell lines during exposure to fluorescent DNA probes. For the Intact-Surface-FISH assay targeting surface RNAs, live primary cells or cell lines were used without fixation or permeabilization to preserve membrane integrity. 1 mmol/L ATP, rather than formamide, was utilized to wash away non-specific oligo binding due to its non-denaturing properties, ensuring cell viability and allowing for subsequent confocal imaging and flow cytometry analysis with live cells.

We confirmed membrane integrity through transmission-through-dye (TTD) microscopic analysis, as described in previous studies (Huang *et al.*, 2020; Supplementary Methods and Fig. 1B). The membrane-impermeant quencher acid blue 9 (AB9) did not quench the fluorescence of cells stained with CellTracker Orange in either the control or the “Intact-Surface-FISH”-treated cells (Fig. 1A, upper and middle panels). However, in permeabilized cells treated with 0.2% Triton, the AB9 quencher penetrated, reducing fluorescence signals (Fig. 1B, bottom panel). These results confirm that the “Intact-Surface-FISH” procedure maintains cell membrane integrity.

Incubation of the Cy3-20N (random A/T/C/G) probe with live HeLa and HEK 293T cells revealed a clear fluorescent signal localized to the cell membrane through the Intact-Surface-FISH assay. The use of random 20N DNA probes, rather than shorter probes, was intended to ensure robust binding between complementary DNA probes and surface RNAs, thereby facilitating effective imaging. This signal was susceptible to RNase digestion (Fig. 1C). Application of Intact-Surface-FISH to live human umbilical cord blood mononuclear cells (hUCB-MNCs), followed by flow cytometry analysis (Fig. 1D and 1E) and confocal imaging (Fig. 1F), further supported the presence of cell surface-anchored RNA, demonstrating the feasibility of our approach. These findings corroborate recent observations of surface RNA association with cultured cell outer membranes (Flynn *et al.*, 2021; Huang *et al.*, 2020) and validate our intact imaging method.

### “AMOUR” enables *in situ* amplification of outer membrane surface RNAs

Despite the successful identification of surface RNA using the high-throughput method “Surface-seq” (Huang *et al.*, 2020), challenges remain in analyzing rare cell populations. Although the spatial imaging approach “ARPLA” (Ma *et al.*, 2024) is highly sensitive and provides detailed spatial information, it is specialized for imaging a specific type of surface RNA rather than multiple surface RNAs in a high-throughput manner. Therefore, our aim is to develop a non-disruptive technique that facilitates comprehensive and accurate profiling of global surface RNA residing on the plasma membrane, particularly focusing on rare primary cell populations. Building on promising flow cytometry and imaging observations (Fig. 1C–F), we introduce a new method, termed *in-situ* amplification of outer membrane surface RNA (AMOUR), designed to profile surface RNA species while preserving cell membrane integrity. AMOUR harnesses T7 RNA polymerase’s capability to utilize both DNA and RNA as templates for *in vitro* transcription (Cazenave and Uhlenbeck, 1994), integrating approaches we previously developed in Jump-seq (Hu *et al.*, 2019).

**Figure 2.**
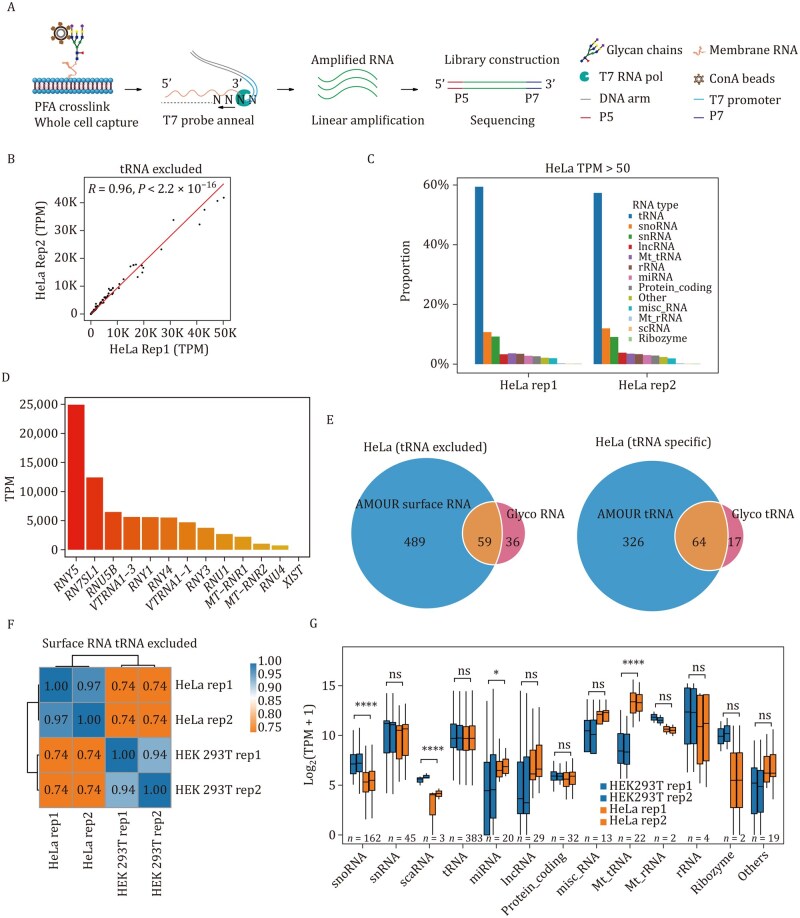
**Detection of surface RNAs via AMOUR strategy on the outer membrane of HeLa and HEK 293T cells**. (A) Schematic representation detailing the workflow of the *in-situ* amplification of outer membrane surface RNA (AMOUR) technique. Fixed cells are immobilized using ConA beads, followed by hybridization with T7 probes and amplification using T7 RNA polymerase. The resulting amplified RNA undergoes library construction and subsequent sequencing. (B) Pearson correlation analysis of two independent replicates of AMOUR datasets for cultured HeLa cells, excluding tRNA. (C) Bar Plot showing the proportions of transcript types (only those with TPM >50) identified using AMOUR in HeLa cells, excluding tRNA. (D) Bar plot showcasing the transcripts per million (TPM) values of select representative surface RNA molecules identified in HeLa cells. (E) Venn plot demonstrating the overlap between surface RNA detected via AMOUR and glycoRNAs reported by Ryan A. Flynn *et al.* (*Cell*, 2021), excluding tRNA (left panel) or tRNA-specific (right panel). (F) Heatmap depicting the correlation of surface RNAs identified in HeLa and HEK 293T cells using AMOUR. (G) Box plots display TPM expression values across various RNA categories in the HeLa and HEK 293T cells identified with AMOUR. Transcripts with a TPM value greater than 50 in at least one biological sample were included in the analysis. The number of transcripts in each RNA category is shown at the bottom. Data are presented as the mean ± SD and were analyzed using an unpaired two-tailed Student’s *t*-test; ns: not significant. *: *p* < 0.05, ****: *p*< 0.0001.

In the context of AMOUR (Fig. 2A; Supplementary Methods), it is crucial to maintain gentle procedures, particularly during *in-situ* amplification, to preserve cell membrane integrity. Live cultured cells or primary cells (with viability no less than 95%) were harvested without trypsin treatment. Following gentle washes with 1× Phosphate Buffered Saline (PBS), cells were pelleted by centrifuging at 500×*g* at 25°C for 5 min. Then, the cells were fixed using 4% paraformaldehyde in PBS for 15 min at 25°C to stabilize the cell membrane. Cells were then immobilized onto magnetic ConA beads, and a double-stranded DNA probe (T7N9 oligo) containing a T7 promoter sequence with protruding nine N bases (random A/T/C/G) was annealed (Supplementary Document S1). We selected random nine-nucleotide sequences, rather than longer sequences, for annealing with surface RNAs to provide versatile binding sites that encompass a broad range of surface RNA regions, in accordance with the single-cell bisulfite sequencing strategy (Smallwood *et al.*, 2014). Excess DNA probes were washed away using 1× PBS containing 1 mmol/L ATP before *in situ* amplification. T7 RNA polymerase (detergent-free), along with Nucleoside triphosphates (NTP), was introduced (final reaction condition: 0.5× PBS and 0.5× commercial reaction buffer to maintain acceptable osmotic pressure) to initiate *in-situ* amplification at 37°C. The T7 RNA polymerase identifies the T7 promoter in the dsDNA probe, transcribing RNA along it, and “jumping” onto surface RNAs upon encountering the RNA-random N hybrid, thereby continuing transcription utilizing surface RNAs as templates. This process yields amplified RNA ready for subsequent reverse transcription with Moloney Murine Leukemia Virus (M-MLV) Reverse Transcriptase, library construction, and high-throughput sequencing (Supplementary Methods). Unlike the Intact-Surface-FISH assay, which utilizes live cells for coupling with subsequent live cell flow cytometry analysis, the AMOUR method employs fixed cells. This approach is essential for working with rare primary cells, as live cells exhibit poor binding to ConA beads due to the instability of their cell states. Fixed cells are used to ensure efficient capture for surface RNA profiling, minimizing significant cell loss caused by repetitive washing and centrifugation steps.

### Validation of AMOUR strategy

We initially examined the impact of AMOUR treatment on cell membrane integrity. Microscopic analysis following AMOUR treatment with TTD (Supplementary Methods) revealed that cells retained resistance to the quencher and exhibited fluorescence intensities comparable to the control group, in contrast to cells treated with 0.2% Triton X-100, which showed significant damage (Fig. S1A). Control experiments indicate that neither the Alexa Fluor 647-GAPDH antibody, Cy3-GAPDH oligo, nor the Alexa Fluor 647-T7 RNA polymerase penetrates fixed cells under AMOUR conditions (Fig. S1B–D). Significant signals of Cy3 or Alexa Fluor 647 were observed only in cells permeabilized with 0.2% Triton X-100. Notably, mild permeabilization using 0.001% or 0.01% Triton X-100 resulted in minimal leakage of Alexa Fluor 647 T7 RNA polymerase, with substantial leakage occurring only following thorough permeabilization at 0.2% Triton X-100 (Fig. S1D). Imaging data confirmed that complete permeabilization of fixed cells, rather than minimal damage, induces leakage of macromolecules like T7N9 oligos or T7 RNA polymerase. Since the AMOUR assay uses cells with ≥95% viability and preserves membrane integrity, these findings underscore the method’s practical applicability.

We then assessed the effectiveness of the AMOUR strategy for *in situ* amplification using a 325 nt biotinylated model RNA immobilized on streptavidin beads, demonstrating robust amplification efficiency and reads coverage (Fig. S2A). We further applied AMOUR amplification to lysed HeLa cells, as well as to HeLa cells pre-treated with RNase A and T1 in their live states prior to fixation, to serve as additional controls. The results revealed significant differences compared to the standard HeLa AMOUR assay (Fig. S2B). Notably, amplifying just 3% of the cell lysate (Supplementary Methods) produced much stronger signals than the standard AMOUR assay, while the RNase pre-treated group exhibited a marked reduction in surface RNA signals (Fig. S2C). We noted that several surface RNAs, including *RNVU1* and *RNU6*, retained strong signals despite RNase treatment. We observed that these specific RNAs are highly structured, which protects them from RNase degradation and leads to their over-amplification by T7 RNA polymerase, followed by sequencing. Transcripts enriched in the RNase-treated group displayed a greater propensity to form stable secondary structures compared to both the 3% cell lysate and AMOUR control groups (Fig. S2D). Consistent with this observation, analysis of the sequence context of these enriched transcripts revealed a significant increase in G/C content (Fig. S2E). Since TPM (transcripts per kilobase per million mapped reads) represents the relative abundance of a transcript within a population of sequenced transcripts (Zhao *et al.*, 2020), these highly structured surface RNAs, which are resistant to RNase, exhibit strong signals during sequencing.

Transcripts identified by AMOUR with a TPM value greater than 50 are considered positive hits. TPM values for snoRNA, scaRNA, rRNA, and ribozyme in the AMOUR control group are significantly lower than those in the 3% cell lysate group, while mitochondrial tRNAs are significantly enriched in the AMOUR group (Fig. S2F). The TPM values of representative small RNAs are higher than those of protein-coding RNAs identified by AMOUR (Fig. S2G). Mitochondrial tRNAs, including *MT-TA*, *MT-TN*, and small RNAs such as *RNY1*, *RNY4*, and *VTRNA1-1*, are predominantly enriched in the AMOUR control group (Fig. S2G). In contrast, *RNY3*, *RNY5*, and all representative protein-coding RNAs—such as *H3C1*, *H4C3*, *RANBP1*, *RPL13*, *RPL36A*, *RPL41*, and *TMEM107*—are enriched in the cell lysate group (Fig. S2G). The TPM values of all representative RNAs exhibit a marked reduction in the RNase pre-treated group, followed by AMOUR. Notably, mitochondrial tRNAs retain substantial TPM values in the RNase pre-treated group, likely due to their stable secondary structures (Fig. S2G, left panel). Quantitative analysis further reveals that the surface RNAs identified by AMOUR exhibit a distinct pattern, rather than representing leakage from the intracellular compartment. The prevalent presence of surface tRNAs detected aligns well with previous reports indicating that tRNAs are highly glycosylated and displayed on the cell surface (Flynn *et al.*, 2021; Xie *et al.*, 2024). Previous research has shown that human tRNA^Sec^ associates with HeLa membranes, cell lipid liposomes, and synthetic lipid bilayers (Janas *et al.*, 2012), consistent with our findings. This supports the need for further investigation into the role of surface tRNAs.

### Surface RNAs on the outer membrane of HeLa and HEK 293T cells

Surface RNAs from the outer membrane of HeLa cells were profiled using the AMOUR strategy (Table S1), revealing consistent patterns across two biological replicates (Fig. 2B). This consistency underscores the reliability and stability of our approach. Notably, the majority of surface RNAs tethered to the outer membrane consist of tRNAs and other non-coding RNAs, including small nucleolar RNAs (snoRNA) and small nuclear RNAs (snRNA), with protein-coding RNAs forming a minority (Fig. 2C; Table S1).

Representative examples illustrating different types of surface RNAs (Fig. 2D) suggest potential diverse roles. Among these, *RN7SL3* RNA stands out as a key component of the signal recognition particle (SRP), responsible for the transport of membrane proteins to the plasma membrane, an association consistent with its surface localization (Akopian *et al.*, 2013). Furthermore, the interaction of *VTRNA1-1* with P62 and its implicated role in autophagy inhibition prompt compelling inquiries into its function when localized on the cell surface (Horos *et al.*, 2019). Additionally, the presence of mitochondrial rRNA *MTRNR1*/*2* and the lncRNA *XIST*, known for initiating X chromosome silencing, is surprising. We were particularly intrigued by several snRNAs, including *U1*/*2*/*4*/*5*/*6*, and Y family RNAs (*RNY1*/*3*/*4*/*5*), given the significant role of spliceosomal RNAs in constituting the Sm antigen (Ahlin *et al.*, 2012) and the binding of Y RNAs to systemic lupus erythematosus (SLE) canonical autoantibodies Sjögren's syndrome type A (SSA) and Sjögren's syndrome type B (SSB) antigens Ro60 and La (Boccitto and Wolin, 2019; Reed *et al.*, 2013), respectively. Surface RNAs identified through the AMOUR strategy encompass the majority of previously reported glycoRNAs (Flynn *et al.*, 2021; Fig. 2E).

The surface RNA profile of HEK 293T cells closely resembles that of HeLa cells (Fig. 2F). Quantitative analysis shows that the TPM values of HEK 293T surface snoRNA, scaRNA, and miRNA are higher than that of HeLa surface RNAs, while surface mitochondria tRNAs tend to be more abundant in HeLa cells (Fig. 2G).

### Validation of AMOUR results

To ensure both sensitivity and specificity, we employed a complementary approach for surface RNA profiling alongside AMOUR. This method incorporates a wheat germ agglutinin (WGA) pull-down assay (WGA-Pd, Fig. S3A), in which Biotin-WGA is used to capture plasma membranes enriched in N-acetylglucosamine and N-acetylneuraminic acid (sialic acid). This is followed by surface RNA extraction and reverse transcription using M-MLV Reverse Transcriptase. We define surface RNAs as any RNA molecules displayed on the outer cellular membrane, whether glycosylated or not, and regardless of whether they directly or indirectly anchor to the cell surface. Given the widespread use of WGA as a cell membrane marker, we employed Biotin-WGA to selectively pull down all RNAs associated with the cell membrane through glycosylated proteins, glycans, or glycosylated lipids, serving as a comprehensive positive control. A comparison of enriched surface RNAs obtained through WGA and AMOUR reveals a notable consistency (Fig. S3B), supporting the specificity of AMOUR. Considering that we utilized 1 million cells for AMOUR compared to 20 million cells for WGA-Pd during surface RNA identification (Supplementary Methods), AMOUR demonstrates superior sensitivity over WGA-Pd when working with limited primary cell samples. Integrative Genomics Viewer (IGV) tracks displaying the reads coverage of *RNY1*/*3*/*4*/*5* identified by the two approaches further demonstrate that AMOUR and WGA-Pd generate complete coverage (Fig. S3C).

Intriguingly, the read coverage patterns for entire genes of several representative surface RNAs identified by AMOUR in HeLa cells exhibit notable variability (Fig. S3D). Specifically, *RNAY1*/*3*/*4*/*5* show even coverage across the entire gene, while *MTNR2* exhibits a 3′ biased coverage. Spliceosomal RNAs, including *U1*/*4*/*5*, demonstrate coverage concentrated in the middle of the gene, whereas *RN7SL1* displays both 5′ and 3′ biased coverage. We speculate that Y RNAs are likely exposed extracellularly, whereas other transcripts may have regions embedded within transmembrane structures or are located intracellularly.

Leveraging T7 RNA polymerase’s *in-situ* linear amplification and strand displacement capabilities, AMOUR proves to be a highly sensitive method for profiling surface RNA, especially when working with limited primary cell numbers. Notably, the sensitivity and specificity of AMOUR, without disrupting the cell membrane, allow us to map surface RNA landscapes across diverse primary cell types.

### Verification of identified surface RNA molecules with cultured HeLa cells and hUCB-MNCs

Reports have indicated the presence of surface RNAs, notably glycoRNAs, on the plasma membrane (Flynn *et al.*, 2021; Huang *et al.*, 2020). Imaging-based analyses have confirmed the localization of specific surface RNAs on both cultured cell lines (Flynn *et al.*, 2021; Huang *et al.*, 2020; Ma *et al.*, 2024) and peripheral blood mononuclear cells (PBMCs; Huang *et al.*, 2020). To validate the membrane localization of previously identified surface RNAs (Flynn *et al.*, 2021) and those identified via AMOUR, we employed the self-developed Intact-Surface-FISH assay (Supplementary Methods and Fig. 1A). Confocal imaging data clearly demonstrate the localization of small RNA *RNY5* and mt-rRNA *MT-RNR1*/*2* on the surface of cultured HeLa cells (Fig. S4A), labeled with complementary Cy3 probes (Supplementary Document S1). We designed the oligonucleotides to be 35–40 nucleotides in length, with a GC content ranging from 40% to 60%. Sequences containing continuous CG trinucleotides were excluded. For small RNA targets, one to two oligonucleotide sequences were designed to cover the entire transcript. For longer transcripts, two to three oligonucleotide sequences were designed to cover two to three exons (Supplementary Document S1). Using flow cytometry, we quantified the proportion of live hUCB-MNCs displaying several highlighted surface RNAs, including small RNA *RNY5*, mitochondrial rRNAs *MT-RNR1*/*2*, spliceosomal RNA *U5*, and lncRNA *XIST* (Fig. S4B). These representative surface RNAs, which encompass various RNA types, were selected due to their documented essential biological functions. We further confirmed the colocalization of these surface RNAs with the plasma membrane in primary hUCB-MNCs (Fig. S4C). Notably, Cy3-probes complementary to lambda DNA and GAPDH showed no signal, further reinforcing the assay’s specificity. Our flow cytometry analysis and imaging results unequivocally confirm the membranal localization of Y family RNA *Y5*, spliceosomal snRNA *U5*, mt-rRNAs, and lncRNA *XIST*. Notably, primary hUCB-MNCs exhibit a broader variety of surface RNA types compared to cultured HeLa cells (Fig. S4A–C), potentially due to their greater cellular diversity.

**Figure 3.**
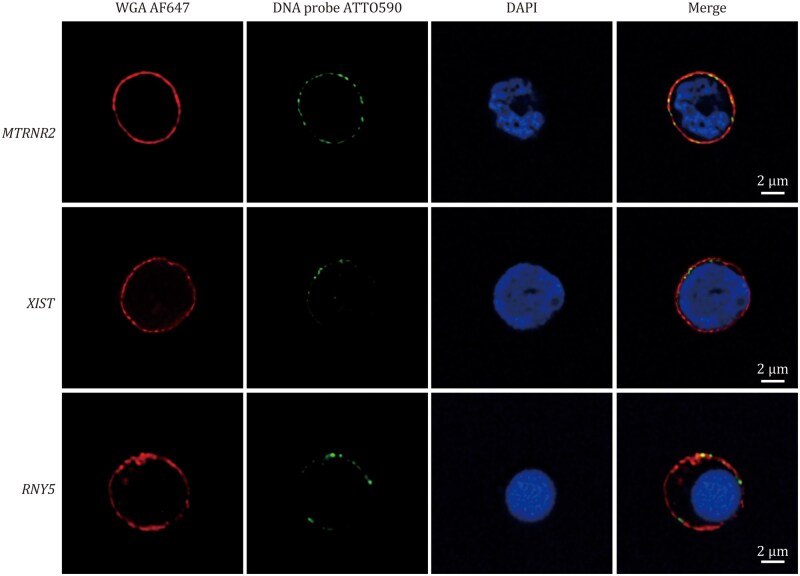
**Co-imaging of surface RNAs *MTRNR2*, *XIST*, and *RNY5* (green) and plasma membrane tracker WGA (red) in hUCB-MNCs with Intact-Surface-FISH, utilizing stimulated emission depletion (STED)**  **microscopy STEDYCON**. For additional visualization, refer to the 3D imaging results in Videos S1–3.

Co-imaging of representative surface RNAs *MTRNR2*, *XIST*, and *RNY5* with the plasma membrane tracker WGA in hUCB-MNCs at nanometer scale (Fig. 3; Videos S1–3) using Intact-Surface-FISH clearly demonstrates their localization to the membrane rather than intracellularly.

**Figure 4.**
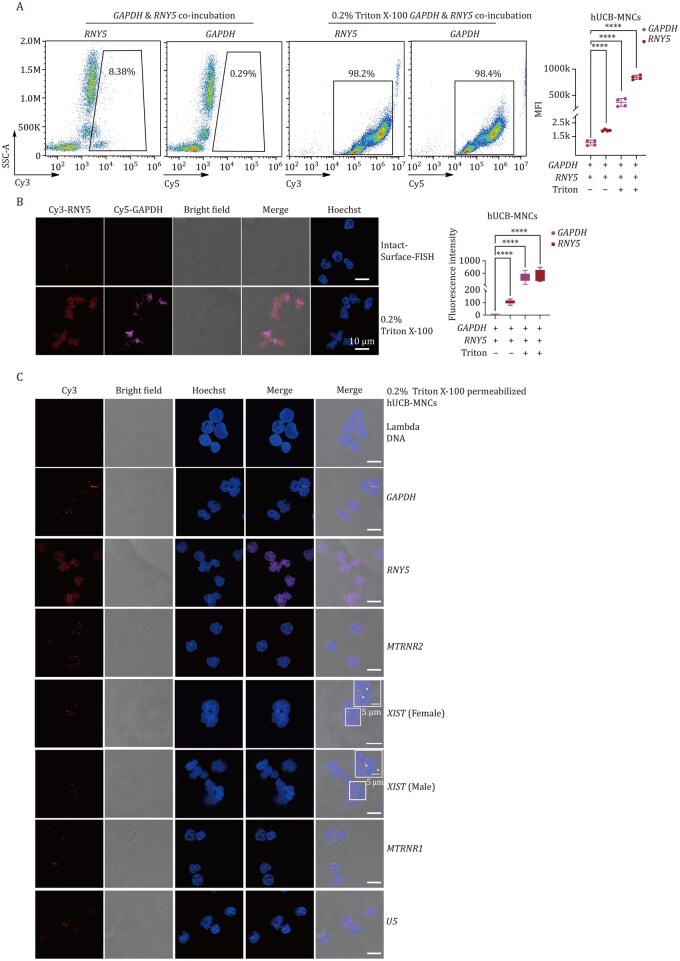
**Validation of surface RNA localization using Intact-Surface-FISH**. (A) Fluorescence co-labeling of live or fixed and permeabilized hUCB-MNCs with Cy5-DNA probes complementary to mRNA *GAPDH* and Cy3-DNA probes complementary to *RNY5*, using Intact-Surface-FISH. The gated region indicates the cell population displaying positive Cy3 and Cy5 signals. Quantitative analysis of the mean fluorescence intensity within the Cy3/Cy5-positive region is provided for each treatment. Data represent four independent experiments. (B) Confocal co-imaging of live or fixed and permeabilized hUCB-MNCs with Cy5-DNA probes complementary to mRNA *GAPDH* and Cy3-DNA probes complementary to *RNY5*, using Intact-Surface-FISH or conventional RNA-FISH. Quantitative analysis of the fluorescence intensity per image is provided for each treatment. (C) Confocal co-imaging of fixed and permeabilized hUCB-MNCs using Cy3-DNA probes complementary to representative RNAs, performed with conventional RNA-FISH. Quantitative analysis of the fluorescence intensity per image is provided for each treatment. All imaging experiments were conducted independently in triplicate, with similar results observed across all repetitions.

To further validate membrane integrity during the Intact-Surface-FISH assay, we co-incubated Cy3-*Y5* and Cy5-*GAPDH* and performed Intact-Surface-FISH on both live and fixed, permeabilized hUCB-MNCs. Flow cytometry (Fig. 4A) and confocal imaging (Fig. 4B) revealed that small RNA *Y5*, rather than mRNA *GAPDH*, is prominently displayed on the surface of live hUCB-MNCs. The surface fluorescence signals of these representative RNAs distinctly differ from the intracellular signals observed after fixation and permeabilization utilizing conventional RNA-FISH (Fig. 4A–C). Please note that for imaging RNAs localized within the nucleus after fixation and permeabilization, a prolonged incubation period of no less than 6 h at 37°C is essential to ensure thorough hybridization of Cy3-DNA probes with nuclear RNAs (Supplementary Methods). The negligible fluorescent signal of Lambda DNA in both intact and permeabilized cells confirms the specificity of the imaging (Figs. 4C; Fig. S4A–C). The labeling efficiency of *U5* RNA, whether using Intact-Surface-FISH (Fig. S4A–C) or conventional FISH (Fig. 4C), is lower compared to other detected RNAs due to its highly folded structures. Co-imaging of *MT-TA*, *VTRNA1-1*, *RNY1*/*3*/*4*, and WGA on the surface of hUCB-MNCs using Intact-Surface-FISH also demonstrated clear surface localization (Fig. S5A), in contrast to the intracellular signals observed after fixation and permeabilization using conventional RNA-FISH (Fig. S5B).

Our validation of representative surface RNAs highlights the practicality of the AMOUR strategy. Notably, no current approach—whether *in situ* amplification using intact cells like AMOUR, pull-down with Biotin-WGA, or biotinylation of all surface RNA molecules followed by pull-down (Perr *et al.*, 2025)—can fully eliminate the risk of intracellular RNA contamination. Instead, all existing methods strive to enrich potential surface RNA candidates as accurately as possible. AMOUR offers a practical tool for identifying previously uncharacterized, abundantly enriched surface RNA species in limited primary cell populations, while Intact-Surface-FISH enables the visualization and quantification of specific surface RNA molecules on live cell membranes across diverse biological contexts. The integration of AMOUR with Intact-Surface-FISH enables comprehensive screening, improves accuracy, supports statistical analysis, and establishes a foundation for subsequent functional studies of surface RNAs in primary cells.

### Intracellular RNAs are transported and presented on the cell surface

Labeling the surface RNAs of live hUCB-MNCs with Cy3-N20 using Intact-Surface-FISH revealed that surface RNAs are more abundant on the surface of monocytes than on B cells and T cells (Fig. S6A). Supplementation with Brefeldin A, a known inhibitor of membrane trafficking and exocytosis, significantly reduced the surface RNA signal on live hUCB-MNC monocytes, as demonstrated by both flow cytometry analysis (Fig. S6B) and confocal microscopy imaging (Fig. S6C). These findings suggest that at least a portion of surface RNA may be transported to the cell membrane via vesicle trafficking. Further studies are required to confirm this hypothesis.

### Comprehensive mapping of surface RNAs residing on the plasma membrane of mammalian blood cells

To explore the functional significance of surface RNAs, we utilized the AMOUR approach to characterize these molecules present on the outer membrane of *Homo sapiens* and *Mus musculus* blood cells. To investigate the general characteristics and abundance of surface RNAs on human blood cells, we utilized neonate hUCB-MNCs rather than adult PBMCs, due to the higher abundance of hematopoietic stem and progenitor cells (HSPCs) in neonate hUCB-MNCs and their significantly lower biological variability compared to PBMCs isolated from adult donors. Our investigation encompassed various blood cell types, including B cells, T cells, HSPCs, natural killer (NK) cells, and monocytes. Our approach successfully delineated a detailed landscape of surface RNAs across mammalian blood cells (Tables S2–S5). Correlation analysis demonstrates excellent consistency among the three biological replicates, with Pearson correlation coefficients ranging from 0.84 to 0.99 (Figs. S7 and S8). While we excluded the conserved surface tRNAs, the investigation of the most abundant non-tRNA surface RNAs (top 150) revealed a predominance of non-coding RNAs in both human (Fig. S9A) and mouse species (Fig. S9B). Notably, in the non-tRNA group, snRNA, snoRNA, rRNA, and mt-rRNA made up the largest proportions, with misc-RNA and miRNA also showing substantial representation. Interestingly, ribozyme RNAs were detected on the surface of both human and mouse HSPCs (Fig. S9A and S9B), suggesting their potential biological relevance. Additionally, surface RNA types and their abundance across different cell types within the same species displayed a high degree of similarity (Fig. S9C).

**Figure 5.**
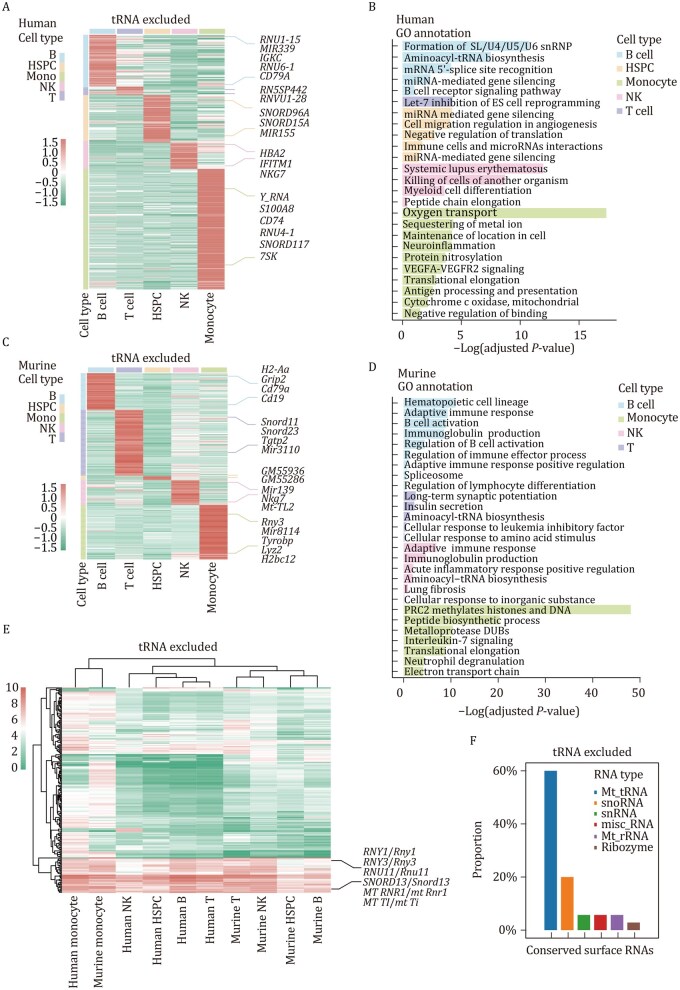
**Surface RNA profiles across diverse human and murine blood cell types**. (A) Heatmap illustrating distinctive surface RNA clusters characterizing various human blood cell types. Each row represents a specific cell type, and columns correspond to individual RNA clusters. Highlighted representative RNA signatures elucidate unique expression patterns across these cell types (excluding tRNA). (B) Gene Ontology (GO) analysis revealing enriched gene functions associated with observed surface RNAs across diverse human blood cell types (excluding tRNA). (C) Heatmap displaying specific surface RNA clusters identified within distinct murine blood cell subsets (excluding tRNA). Highlighted representative RNA signatures emphasize discernible expression patterns within these subsets. (D) GO analysis delineating enriched gene functions linked to surface RNAs identified across diverse murine blood cell types (excluding tRNA). (E) Heatmap spotlighting highly abundant surface RNA clusters conserved across all analyzed blood cell types in both human and mouse (excluding tRNA). Highlighted representative RNA signatures underscore shared expression patterns among these cell types. (F) Bar plot illustrating the RNA types of conserved surface RNAs across all analyzed blood cell types in both human and mouse (excluding tRNA).

Our further analysis focused on relatively abundant non-tRNA surface RNAs in each cell type of both human (Fig. 5A and 5B; Table S2) and mouse (Fig. 5C and 5D; Table S3). Notably, immune cell types exhibited enrichment in inflammation, innate, and adaptive immune-related functions (Fig. 5B and 5D), whereas human HSPCs highlighted miRNA and translation-related functions (Fig. 5B) due to the presence of miRNA and snoRNA on their membrane surfaces. The cell-type-specific surface RNAs provided by our study form a crucial basis for further investigations into biological functions. Representative highly abundant surface RNAs, spanning spliceosomal U RNA, ribosomal RNA, microRNA, mitochondrial tRNA, and protein-coding RNA, were meticulously identified for each distinct human and murine (Fig. 5E) blood cell type. The abundant presence of surface RNAs on human monocytes is consistent with previous report (Huang *et al.*, 2020).

Remarkably, several non-tRNA surface RNA molecules exhibited high expression patterns across all blood cell types in both human and mouse (Fig. 5E; Fig. S9F; Table S4), indicating evolutionarily conserved biological functions. These “housekeeping” surface RNAs included Mt-tRNA, snoRNA, snRNA, misc-RNA, Mt-rRNA, and ribozyme (Fig. 5F), with selected representatives highlighted (Fig. S9F).

We then focused on surface tRNAs that remained conserved across cell types and between human and murine species. The analysis revealed that surface tRNA expression is abundant in all blood cell types (Table S5). We delineated cell-type-specific surface tRNAs in humans (Fig. S9G) and mice (Fig. S9H), as well as those conserved across various cell types and species (Fig. S9I). Notably, both human and mouse HSPCs exhibit a significantly broader spectrum of tRNA types (Fig. S9G and S9H) compared to other cell types, suggesting the putative specialized functional roles of surface tRNAs within the context of HSPCs.

Human UCB T cells exhibit fewer surface RNAs compared to B cells and monocytes (Fig. 5A; Fig. S9G). Total RNA sequencing reveals that the TPM values of transcripts across various RNA categories in T cells are comparable to those in B cells and monocytes (Fig. S9J). This observation suggests that the reduced abundance of surface RNAs in T cells is not attributable to differential gene expression. We hypothesize the presence of specific, yet unidentified, surface transport mechanisms.

## Discussion

In summary, our work significantly strengthens the evidence affirming the presence of surface-anchored RNAs on the outer membrane of primary cells. Our comprehensive portrayal of the surface RNA landscape within mammalian blood cells establishes a robust foundation for further investigations aimed at uncovering the biological implications and functional significance of surface RNAs. The AMOUR method developed in this study enables sensitive profiling of surface RNAs displayed on the cell membranes of primary cells. When combined with Intact-Surface-FISH, followed by imaging and flow cytometry analysis, researchers can validate the spatial distribution of specific surface RNAs and compare their abundance across various cell types.

The methods developed in this study for profiling and imaging surface RNAs, as well as investigating surface RNA-binding proteins, employ live cells with a viability of no less than 95%. Consequently, live cells freshly dissected from tissue are required for surface RNA investigations. This limitation restricts the application of these techniques to frozen samples or formalin-fixed, paraffin-embedded (FFPE) samples, due to the risk of cell rupture and subsequent contamination from intracellular RNA. To address these limitations, it is crucial to develop additional methods that employ specialized strategies for investigating surface RNAs in frozen cells and FFPE samples. Considering that surface RNAs are cell-type-specific, methodologies capable of profiling both intracellular and surface RNAs simultaneously at the single-cell level are highly desirable.

While significant progress has been made in validating and understanding surface RNAs, many aspects of surface RNA biology across various cell types remain unexplored. For instance, Y RNAs are significantly enriched on the surfaces of human immune cells and have been reported to bind with SLE canonical autoantibodies SSA and SSB antigens Ro60 and La (Boccitto and Wolin, 2019; Reed *et al.*, 2013). Investigating the role of Y RNAs in innate and adaptive immunity will be an intriguing direction for future research. Several prevalent long non-coding surface RNAs, such as Mt-rRNA, may harbor significant functions warranting further investigation. U snRNAs (U1, U2, U4, U5, and U6 RNAs), integral components of U ribonucleoproteins (RNPs), are known as Sm antigens in the sera of SLE patients (Migliorini *et al.*, 2005). Autoantibodies targeting aminoacyl-tRNA synthetases are implicated in the pathogenesis of anti-synthetase syndrome (Mahler *et al.*, 2014). *XIST* ribonucleoproteins have recently been reported to promote female sex-biased autoimmunity (Dou *et al.*, 2024). It would be intriguing to investigate the biological functions of these unexpected surface RNAs that are closely associated with autoimmune diseases.

Moving forward, we anticipate a diverse spectrum of roles for surface RNAs within distinct cell types, spanning neurons, germ cells, cancer cells, and beyond. Deciphering the intricate workings of surface RNAs necessitates continued investigation and exploration. Notably, the strategies developed in this study provide a foundational framework that will facilitate further advancements in this field.

## Supplementary Material

pwaf079_Supplementary_Data

## Data Availability

The raw sequencing data generated from human blood cells reported in this paper have been deposited in the Genome Sequence Archive (Genomics, Proteomics & Bioinformatics 2021) in the National Genomics Data Center (Nucleic Acids Res 2022), China National Center for Bioinformation/Beijing Institute of Genomics, Chinese Academy of Sciences (GSA-Human: HRA006257) which are publicly accessible at ngdc.cncb.ac.cn/gsa-human. The sequencing data generated from mouse blood cells and cell lines referenced in this study have been meticulously archived and made publicly available via the Gene Expression Omnibus repository under the accession numbers GSE252429, GSE252682, and GSE262356.

## Code availability

The original code has been deposited at GitHub: github.com/xuchu16/Surface-RNA. For inquiries regarding additional information needed for the reanalysis of the data presented in this paper, interested parties may obtain the requisite details from the lead contact upon request.

## Abbreviations:

AMOUR: *in-situ* amplification of outer membrane surface RNA
AB9: acid blue 9
FISH: fluorescence *in situ* hybridization
hUCB-MNCs: human umbilical cord blood mononuclear cells
HSPCs: hematopoietic stem and progenitor cells
mt-RNA: mitochondrial RNA
miRNA: microRNA
NK cells: natural killer cells
snoRNA: small nucleolar RNA
snRNA: small nuclear RNA
scaRNA: small Cajal body-specific RNA
SRP: signal recognition particle
TTD: transmission-through-dye
TPM: transcripts per kilobase per million mapped reads
WGA: wheat germ agglutinin
