# Supplementary material for: Deciphering the RNA landscapes on mammalian cell surfaces
Source: Protein Cell. 2025 Sep 18;17(2):113–26. doi: 10.1093/procel/pwaf079 (PMC12959771; doi:10.1093/procel/pwaf079)
Supplement: pwaf079_Supplementary_Data [file pwaf079_supplementary_data.zip › PAC-25402-HLL-Supplemental materials.pdf]

SUPPORTING INFORMATION for

Deciphering the RNA Landscapes on Mammalian Cell Surfaces

Xiao Jiang[1#], Chu Xu[1#], Enzhuo Yang[1#], Danhua Xu[3#], Yong Peng[6#], Xue Han[1], Jingwen Si[2,7], Qixin Shao[1], Zhuo Liu[1], Qiuxiao Chen[1], Weizhi He[1], Shuang He[1], Yanhui Xu[1], Chuan He[4,5], Xinxin Huang[3*], Lulu Hu[1,2*]

* Correspondence: Lulu Hu and Xinxin Huang

Email: Lulu Hu: luluhu@fudan.edu.cn and Xinxin Huang: xinxinhuang@fudan.edu.cn

This file includes:

Supporting information text
Figures S1 to S9
Document S1
Legends for Datasets S1 to S5
Legends for Videos S1-S3

Supporting Information Text

Methods

Cell culture

HEK 293T and HeLa cells (purchased from the Cell Bank/Stem Cell Bank of the Chinese Academy of Sciences) were cultured in DMEM (BasalMedia) supplemented with 10% (v/v) fetal bovine serum (FBS, BI). THP-1 and Raji cells (purchased from the Cell Bank of the Chinese Academy of Sciences) was cultured in RPMI-1640 medium (Gibco) supplemented with 2.5 mM glutamine, 10% (v/v) FBS (Gibco), and 100 U mL^{-1} penicillin-streptomycin (Sangon Biotech). The cultures were maintained in a 5% CO_2 incubator at 37 °C.

Detailed flowchart of the AMOUR method

1 × 10^6 live HEK 293T cells (≥ 95% viability) were fixed with 4% PFA in 1 × PBS for 15 min at 25 °C and immobilized with Concanavalin A beads (Con A beads, Bangs Laboratories). The bead-bound cells underwent three washes with AMOUR-wash buffer (PBS, 1 mM ATP, 0.2 U μL^{-1} RNase inhibitor, Vazyme). Subsequently, the beads were resuspended in PBS with RNase Inhibitor (40 U μL^{-1}) and annealed with T7N9 oligo (0.25 μM) (*SI Appendix*, Document S1). The reaction mixture was incubated at 37 °C for 5 min, followed by ramping to 25 °C and a 5-min hold. After removing excess T7N9 oligo, linear amplification was performed using T7 RNA Polymerase (Thermo) at 37°C for 2 hours on a rotating shaker. The resulting RNA was purified using Dynabeads MyOne Silane beads (Thermo) and subjected to library construction following the m^6A-sac-seq technology protocol(Hu et al., 2022; Ge et al., 2023). In brief, amplified RNA underwent repair using PNK (NEB) followed by ligation to the 3' Adaptor (/5rApp/AGATCGGAAGAGCGTCGTG/3Bio/) employing T4 RNA Ligase 2, truncated KQ (NEB). Subsequently, it was annealed with the RT primer (ACACGACGCTCTTCCGATCT) and subjected to reverse transcription utilizing M-MLV Reverse Transcriptase (Promega). The resulting cDNA was ligated to the cDNA Adaptor (/5Phos/NNNNNNAGATCGGAAGAGCACACGTCTG/3SpC3/) with T4 RNA ligase 1, high conc. (NEB). Finally, the ligated cDNA underwent library construction using NEBNext UltraII Q5 master mix (NEB), with PCR cycle optimization determined via qPCR.

Labeling of T7 RNA polymerase and anti-GAPDH antibody with Alexa Fluor 647

Alexa Fluor 647-NHS (10 mM, MCE) in DMSO was incubated with T7 RNA polymerase (Thermo) or anti-GAPDH primary antibody (Santa Cruz) at a molar ratio of Alexa Fluor 647-NHS to protein ≥ 3. The reaction mixture was incubated for 30 minutes in 1 × PBS at 25°C, quenched with 100 mM Tris-HCl (pH 7.5), and purified using Micro BioSpin P-6 columns (Bio-Rad).

AMOUR validation utilizing a 325 nt biotin RNA model

A 325-nucleotide RNA model was synthesized *in vitro* utilizing the T7 High Yield RNA Transcription Kit (Vazyme) and subsequently biotinylated using the Pierce RNA 3' End

Biotinylation Kit (Thermo). The resulting biotinylated RNA model was immobilized onto Dynabead MyOne Streptavidin C1 beads (Thermo) and subjected to AMOUR treatment for analysis of amplification efficiency and reads coverage.

AMOUR validation with lysed and RNase A & T1 pre-treated HEK 293T cells

For RNase A & T1 treatment, 1 × 10^6 live HEK 293T cells (with viability no less than 95%) were incubated with 1 μg μL^{-1} RNase A and 1 U μL^{-1} RNase T1 at 37 °C for 20 minutes in 200 μL of 1 × PBS with 1% BSA, followed by three washes with 1× PBS prior to AMOUR. For cell lysis, 1 × 10^6 live HEK 293T cells (with viability no less than 95%) were lysed in 200 μL of lysis buffer (20 mM Tris pH7.5, 300 mM NaCl, 0.5% Triton X-100, 1 U μL^{-1} RNase inhibitor) at 4 °C for 30 minutes, then diluted with 800 μL of 20 mM Tris pH7.5. Subsequently, 30 μL of the diluted cell lysate (3% of total lysate) was annealed with 1 μL of 12.5 μM of T7N9 oligo and subjected to AMOUR amplification.

Wheat germ agglutinin (WGA) enriched membrane-associated RNA pull-down (WGA-Pd)

10 mM biotin-NHS (MCE) in DMSO was combined with 10 mg mL^{-1} Wheat Germ Agglutinin (WGA) protein (Vector) at a molar ratio of biotin-NHS to WGA ≥ 12. The mixture underwent a 30-minute incubation in 1 × PBS at 25 ℃ and was quenched with 100 mM Tris-HCl pH 7.5, followed by purification with Micro BioSpin P-6 (Bio-Rad). 2 × 10^7 HEK 293T cells (with viability no less than 95%) were collected without trypsin treatment, and blocked with Egg white avidin (Thermo) and yeast tRNA (Thermo). Following buffer removal, 500 µg of biotin-WGA was added to the cells and incubated at 37 ℃ for 15 min. After centrifugation at 500 × g, 4 ℃ for 5 min to eliminate excess biotin-WGA, cells were washed with PBS. Lysis was performed with ice-cold lysis buffer (20 mM HEPES pH 7.5, 150 mM NaCl, 1% DDM, and 0.001% GDN with 0.1% CHS, 200 U mL^{-1} RNase inhibitor, 1 × proteinase inhibitor). Dynabeads MyOne Streptavidin C1 (Thermo Fisher Scientific) were utilized for capturing membrane-associated RNA, extracted using Buffer RLT (Qiagen), and subsequently purified using silane beads. Libraries were constructed following the m^6A-sac-seq technology protocol(Hu et al., 2022; Ge et al., 2023) as delineated in the AMOUR method section above. No linear amplification step was conducted in the WGA-Pd assay.

Intact Surface FISH and conventional RNA FISH

Live primary suspension cells (cord blood mononuclear cells) or THP-1 monocytes were hybridized with 100 ng of Cy3-oligos (Document S1) in a hybridization buffer (1× PBS containing 1% BSA) at 37 °C for 30 min, with Lambda DNA probe serving as a negative control. Following hybridization, cells underwent two washes in a washing buffer (1× PBS supplemented with 1% BSA and 1mM ATP pH = 7.0), followed by one additional 10-minute wash with PBS. Labeled cells were analyzed using flow cytometry (CytoFLEX, Beckman Coulter) or spread on polylysine-treated coverslips, dried at 37 °C for 5 minutes, washed twice with 1× PBS containing 1% BSA, mounted with antifade solution, and sealed for imaging.

Adherent cell lines such as HEK 293T and HeLa were detached before incubation with Cy3-oligoes and the other procedures remain the same as primary suspension cells.

For RNase A and T1 treatment, live cells were incubated with 0.02 μg μL^{-1} RNase A and 1 U μL^{-1}T1 at 37 °C for 30 minutes in 1 × PBS, followed by three washes with 1 × PBS before Intact Surface FISH.

For conventional RNA FISH, cells seeded on polylysine-treated coverslips were fixed with 4% PFA and permeabilized with 0.2% Triton X-100 for 10 minutes. Hybridization was performed using 100 ng of Cy3-oligos (Document S1) in a buffer composed of 1× PBS, 100 μg/mL yeast carrier tRNA, 10% dextran sulfate, 20% formamide, and an RNase inhibitor. The hybridization procedure was conducted at 42°C for 5 minutes followed by 37 °C for 6 hours. Cells were subsequently washed twice in wash buffer (20% formamide, 2× SSC).

For bright field imaging, coverslip-sealed cells were visualized using a Leica SP8 confocal laser scanning microscope. For confocal imaging of surface RNAs and the plasma membrane tracker WGA, an FV4000 Confocal Laser Scanning Microscope (Evident Olympus) was employed. For nanometer-scale imaging, the coverslip-sealed cells were examined using a STED nanoscope (STEDYCON, Abberior). The resulting images were processed using ImageJ.

High-throughput sequencing

Library quality assessment was performed using the Agilent 2000 and the ABI Step One Plus Real-time PCR system. High-throughput sequencing for surface RNA profiling of HEK 293T cells, HSPCs, T cells, B cells, and NK cells via AMOUR was conducted on the Illumina HiSeq 2000 sequencer in paired-end 150 bp mode, performed by Sequanta Technologies Co., Ltd. Sequencing for WGA-Pd, high-throughput sequencing of monocytes by AMOUR, AMOUR validations post-cell lysis and RNase A & T1 treatment with the HEK 293T cells was carried out on the DNB T7 sequencing platform in paired-end 100 bp mode at GenePlus, Beijing, China.

Data processing and analysis

RNA-seq reads underwent preprocessing to eliminate low-quality bases and adapters using Trimmomatic v0.39.(Bolger et al., 2014) Subsequently, duplicate reads sharing identical barcodes were identified and filtered, retaining a single representative read to mitigate PCR duplicates, accomplished via BBMap (39.01). Reads lacking barcodes underwent alignment to the Human reference transcriptome (hg38) using Kallisto (0.48.0)(Bray et al., 2016) and to the genome using STAR (2.7.10b).(Dobin et al., 2013) For STAR alignment, the following parameters were utilized: STAR --runThreadN 40 -c --quantMode TranscriptomeSAM --genomeDir /path/to/genomeDir --readFilesIn /path/to/fq --outFileNamePrefix / --outFilterMultimapNmax 20 --outSAMtype BAM Unsorted Only uniquely mapped reads were retained for subsequent analyses. Transcript per million (TPM) value was obtained from the output of Kallisto. FeatureCounts was adopted for counting raw number of reads as input of DESeq2.(Love et al., 2014) Differential expression

analysis for gene-level quantification employed DESeq2,(Love et al., 2014) applying a significance threshold of adjusted p-value < 0.05.

In our study of surface RNA, additional understanding of functional annotations and pathways related to differentially expressed genes (DEGs) was garnered through Gene Ontology (GO) and Kyoto Encyclopedia of Genes and Genomes (KEGG) enrichment analyses performed via Metascape (https://metascape.org/gp/index.html#/main/step1) (Zhou et al., 2019). GO and KEGG enrichment analyses were conducted using clusterProfiler for mRNA(Wu et al., 2021).

Transmission-through-dye (TTD) imaging of HEK 293T cells

The integrity of HEK 293T cell membranes was evaluated using CellTracker Orange CMRA (Thermo), a membrane-permeable dye, and the membrane-impermeant quencher acid blue 9 (AB9, TCI America), following established protocols(Pelts et al., 2011; Huang et al., 2020). HEK 293T cells were plated on polylysine-treated coverslips and stained with 5 μM CellTracker Orange CMRA for 30 min. Subsequently, cells underwent either AMOUR or Intact Surface RNA FISH treatment, supplemented with 5 mg mL^{-1} AB9 for 10 min, and were subjected to confocal imaging. Control groups with disrupted cell membranes were treated with 0.2% (w/v) Triton for permeabilization.

Brefeldin A-mediated vesicle trafficking inhibition assay

Freshly isolated hUCB-MNCs were cultured in RPMI 1640 medium supplemented with 10% FBS. For Brefeldin A (BFA) treatment, BFA (Selleck) was added to the culture medium at the specified concentration (typically 5 μg mL^{-1}) and incubated for 6 hours. Following treatment, the cells were resuspended in 1× PBS containing 1% BSA and adjusted to a concentration of 1× 10^6 cells per 100 µL. The cells were then incubated with 400 ng of Cy3-labeled N20 oligonucleotides at 37°C for 30 minutes.

After incubation, the cells were centrifuged at 350 × g for 5 minutes at 4°C and washed with 500 µL of 1× PBS. Subsequently, Zombie NIR dye (Biolegend) was diluted 1:200 in 1× PBS, and the cells were resuspended in 100 µL of the diluted dye solution and incubated at 4°C in the dark for 30 minutes. Following this incubation, the cells were washed with 1× PBS containing 1% BSA. To block Fc receptors, the cells were incubated with 5 µL of Human TruStain FcX (Biolegend) at 25°C for 10 minutes. Post-blocking, the cells were stained with antibodies targeting specific cell surface markers, including CD45 (Biolegend; leukocyte common antigen), CD14 (Biolegend; human monocytes), CD3 (Biolegend; T cells), and CD19 (Biolegend; B cells). The antibody staining was performed on ice for 20 minutes. Finally, the cells were washed twice with 1× PBS containing 1% BSA to remove any unbound antibodies. The labeled cells were analyzed via flow cytometry using a CytoFLEX instrument (Beckman Coulter). Flow cytometry data were processed and visualized using FlowJo software.

Primary cell isolation

Human hematopoietic stem and progenitor cells (HSPCs), B cells, T cells, NK cells, and monocytes were isolated from human umbilical cord blood (hUCB) samples. Mononuclear

cells were obtained via density gradient centrifugation using Ficoll-Paque Plus (GE Healthcare). Primary cell isolation utilized specific kits: the human CD34 MicroBead Kit (Miltenyi Biotec, 130-046-703) for HSPCs, the human CD19 B cell isolation Kit (Miltenyi Biotec, 130-050-301) for B cells, the human NK cell isolation Kit (Miltenyi Biotec, 130-092-657) for NK cells, the human CD8 T cell isolation Kit (Miltenyi Biotec, 130-096-495) for CD8 T cells, and the human monocyte isolation Kit (Miltenyi Biotec, 130-096-537) for monocytes.

Murine HSPCs and monocytes were isolated from C57BL/6 mouse bone marrow cells. Bone marrow cells were collected by flushing the femur with PBS, followed by filtration through a 30 µm nylon mesh (Miltenyi Biotec Pre-Separation Filters, 130-041-407) to eliminate cell clumps. Primary cell isolation used the mouse lineage cell depletion Kit (Miltenyi Biotec, 130-110-470) for HSPCs and the mouse monocyte isolation Kit (Miltenyi Biotec, 130-100-629) for monocytes.

Murine B cells, T cells, and NK cells were isolated from C57BL/6 mouse spleen cells. The spleen was isolated, and a single-cell suspension was obtained by passing it through a 100-µm cell strainer. Primary cell isolation utilized specific kits: the B cell isolation Kit (Miltenyi Biotec, 130-90-862) for B cells, the CD8 T cell isolation Kit (Miltenyi Biotec, 130-104-075) for CD8 T cells, and the NK cell isolation Kit (Miltenyi Biotec, 130-115-818) for NK cells. Red blood cell lysis buffer was applied to eliminate red blood cells in all samples.

For AMOUR detection, 1 × 10^6 human or mouse B cells, T cells, NK cells, monocytes, and HSPCs were employed.

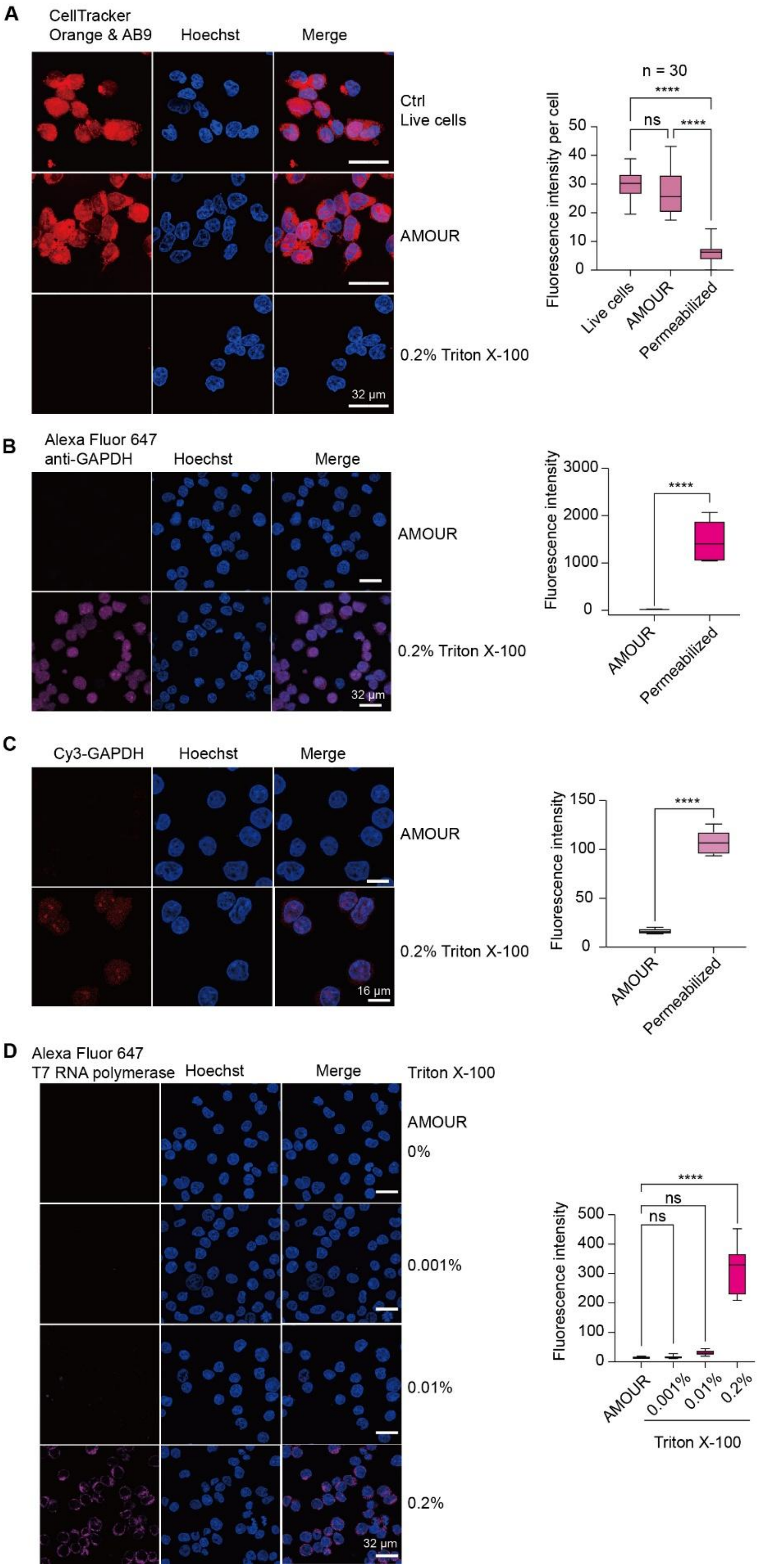
A
CellTracker Orange & AB9
Hoechst
Merge
Ctrl Live cells
AMOUR
0.2% Triton X-100
32 μm
n = 30
****
ns
****
Fluorescence intensity per cell
50
40
30
20
10
0
Live cells
AMOUR
Permeabilized
B
Alexa Fluor 647 anti-GAPDH
Hoechst
Merge
AMOUR
0.2% Triton X-100
32 μm
Fluorescence intensity
3000
2000
1000
0
****
AMOUR
Permeabilized
C
Cy3-GAPDH
Hoechst
Merge
AMOUR
0.2% Triton X-100
16 μm
Fluorescence intensity
150
100
50
0
****
AMOUR
Permeabilized
D
Alexa Fluor 647 T7 RNA polymerase
Hoechst
Merge
Triton X-100
AMOUR 0%
0.001%
0.01%
0.2%
32 μm
Fluorescence intensity
500
400
300
200
100
0
****
ns
ns
AMOUR
0.001%
0.01%
0.2%
Triton X-100

Figure. S1. Validation of the AMOUR strategy in HEK 293T cells using confocal imaging. (A) Transmission-through-dye confocal imaging for HEK 293T cells under different treatments: live cell control group (upper panel), AMOUR-treated group (middle panel), and 0.2% Triton X-100 permeabilized group (bottom panel). Cells with intact membranes show bright fluorescence signals, while cells with damaged membranes exhibit quenched fluorescence signals (left panel). Box plots illustrate the mean fluorescence intensity per cell for the control group, AMOUR-treated group, and the group permeabilized with 0.2% Triton X-100 prior to AMOUR treatment (right panel). (B) Confocal imaging of Alexa Fluor 647-labeled primary GAPDH antibody staining in HEK 293T cells under different treatments (left panel): AMOUR-treated group (upper panel) and 0.2% Triton X-100 permeabilized group (bottom panel). Box plots (right panel) display the mean fluorescence intensity per image for the AMOUR-treated group, and the group permeabilized with 0.2% Triton X-100 prior to AMOUR treatment. (C) Confocal imaging of Cy3-labeled DNA probes complementary to GAPDH mRNA entry into HEK 293T cells under different treatments (left panel): AMOUR-treated group (upper panel), and the group permeabilized with 0.2% Triton X-100 prior to AMOUR treatment (bottom panel). Box plots (right panel) show the mean fluorescence intensity per image for the AMOUR-treated group, and the group permeabilized with 0.2% Triton X-100 prior to AMOUR treatment. (D) Confocal imaging of Alexa Fluor 647-labeled T7 RNA polymerase entry into HEK 293T cells under different treatments (left panel): AMOUR-treated group and Triton X-100 permeabilized groups at concentrations of 0.001%, 0.01%, and 0.2% prior to AMOUR treatment. Box plots (right panel) depict the mean fluorescence intensity per image for the AMOUR-treated group, and the group permeabilized with Triton X-100 prior to AMOUR treatment. The imaging experiments were repeated three times independently with similar results. Data are shown as mean ± s.d., and analyzed using an unpaired two-tailed Student's t-test; ns: not significant; ****: $p < 0.0001$.

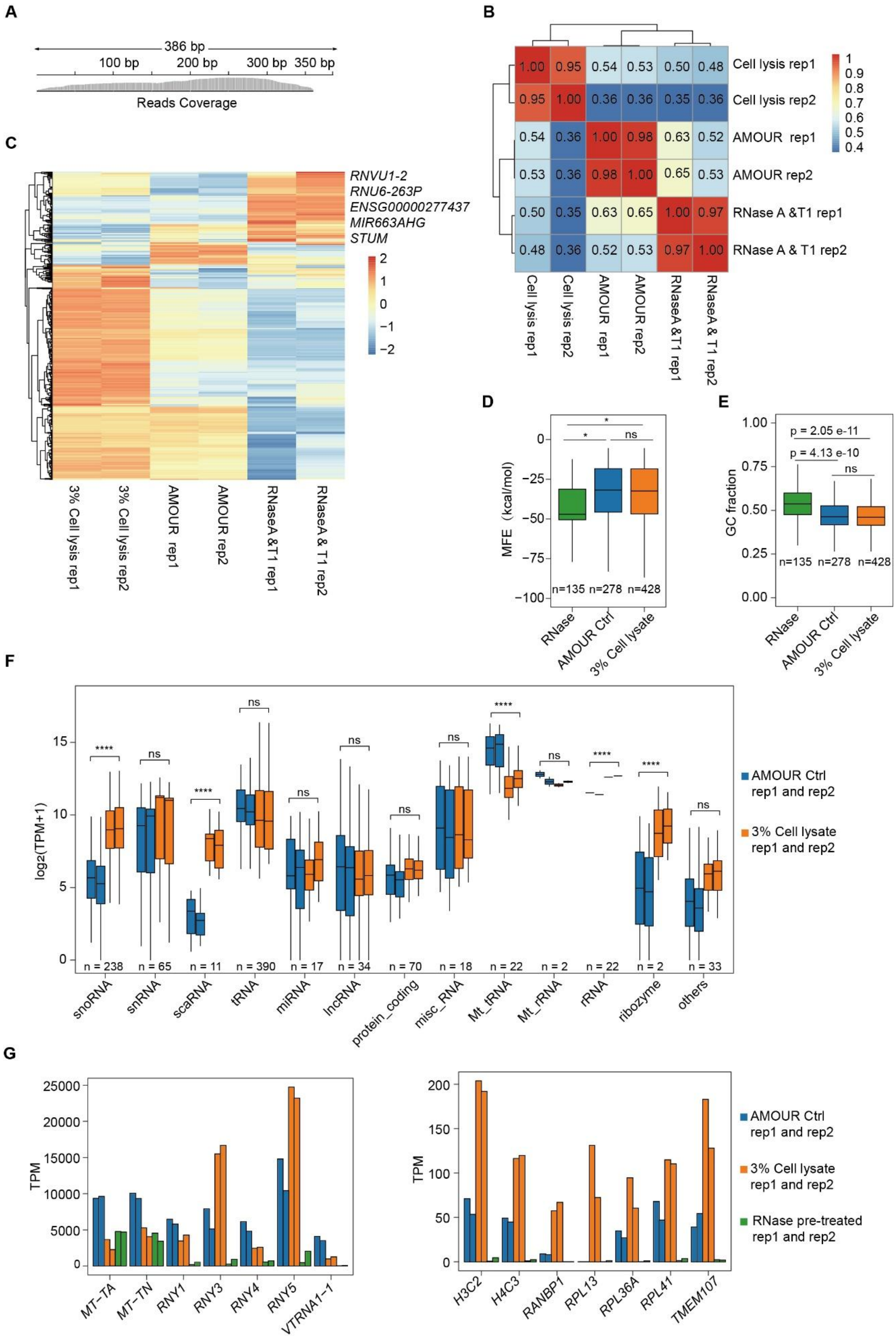
A
386 bp
100 bp
200 bp
300 bp
350 bp
Reads Coverage
B
Cell lysis rep1
Cell lysis rep2
AMOUR rep1
AMOUR rep2
RNase A &T1 rep1
RNase A & T1 rep2
1.00 0.95 0.54 0.53 0.50 0.48
0.95 1.00 0.36 0.36 0.35 0.36
0.54 0.36 1.00 0.98 0.63 0.52
0.53 0.36 0.98 1.00 0.65 0.53
0.50 0.35 0.63 0.65 1.00 0.97
0.48 0.36 0.52 0.53 0.97 1.00
RNaseA &T1 rep1
RNaseA & T1 rep2
C
RNVU1-2
RNU6-263P
ENSG00000277437
MIR663AHG
STUM
3% Cell lysis rep1
3% Cell lysis rep2
AMOUR rep1
AMOUR rep2
RNaseA &T1 rep1
RNaseA & T1 rep2
D
MFE (kcal/mol)
n=135 n=278 n=428
RNase
AMOUR Ctrl
3% Cell lysate
E
GC fraction
p = 2.05 e-11
p = 4.13 e-10
ns
F
log2(TPM+1)
snoRNA
snRNA
scaRNA
tRNA
miRNA
lncRNA
protein_coding
misc_RNA
Mt_tRNA
Mt_rRNA
rRNA
ribozyme
others
n = 238
n = 65
n = 11
n = 390
n = 17
n = 34
n = 70
n = 18
n = 22
n = 2
n = 22
n = 2
n = 33
AMOUR Ctrl rep1 and rep2
3% Cell lysate rep1 and rep2
G
TPM
MT-TA
MT-TN
RNY1
RNY3
RNY4
RNY5
VTRNA1-1
H3C2
H4C3
RANBP1
RPL13
RPL36A
RPL41
TMEM107
RNase pre-treated rep1 and rep2

Figure. S2. Validation of the AMOUR strategy in HEK 293T cells using high-throughput sequencing. (A) Reads coverage of a 325 nt RNA model amplified utilizing AMOUR strategy. (B) Heatmap showing the correlation among specific RNAs identified by AMOUR with 3% HEK 293T cell lysate, AMOUR control, and RNase A & T1 treatment prior to AMOUR in HEK 293T cells (These datasets, generated using the AMOUR technique following various treatments, are intended specifically for method validation. They are distinct from the datasets derived from HEK293T cells presented in Fig. 1 and Fig. S3). (C) Heatmap displaying specific RNAs identified by AMOUR with 3% HEK 293T cell lysate, AMOUR control, and RNase A & T1 treatment prior to AMOUR in HEK 293T cells. (D) The predicted minimum folding free energy (MFE) is plotted for transcripts enriched following RNase A and RNase T1 treatment prior to AMOUR in HEK 293T cells, AMOUR control, and 3% HEK 293T cell lysate groups. The number of transcripts in each group is provided at the bottom. (E) Box plots depict the GC content of transcripts enriched following RNase A and RNase T1 treatment prior to AMOUR in HEK 293T cells, AMOUR control, and 3% HEK 293T cell lysate groups. The number of transcripts in each group is provided at the bottom. (F) Box plots display TPM (transcripts per million) expression values across various RNA categories in the AMOUR control and 3% HEK 293T cell lysate groups. Transcripts with a TPM value greater than 50 in at least one biological sample were included in the analysis. The number of transcripts in each RNA category is shown at the bottom. (G) Box plots showing TPM expression values of representative transcripts identified in the AMOUR control, 3% HEK 293T cell lysate, and RNase A and RNase T1 treatment prior to AMOUR groups. Data are presented as the mean ± standard deviation (s.d.) and were analyzed using an unpaired two-tailed Student's t-test; ns, not significant; *: $p < 0.05$, t; ****, $p < 0.0001$.

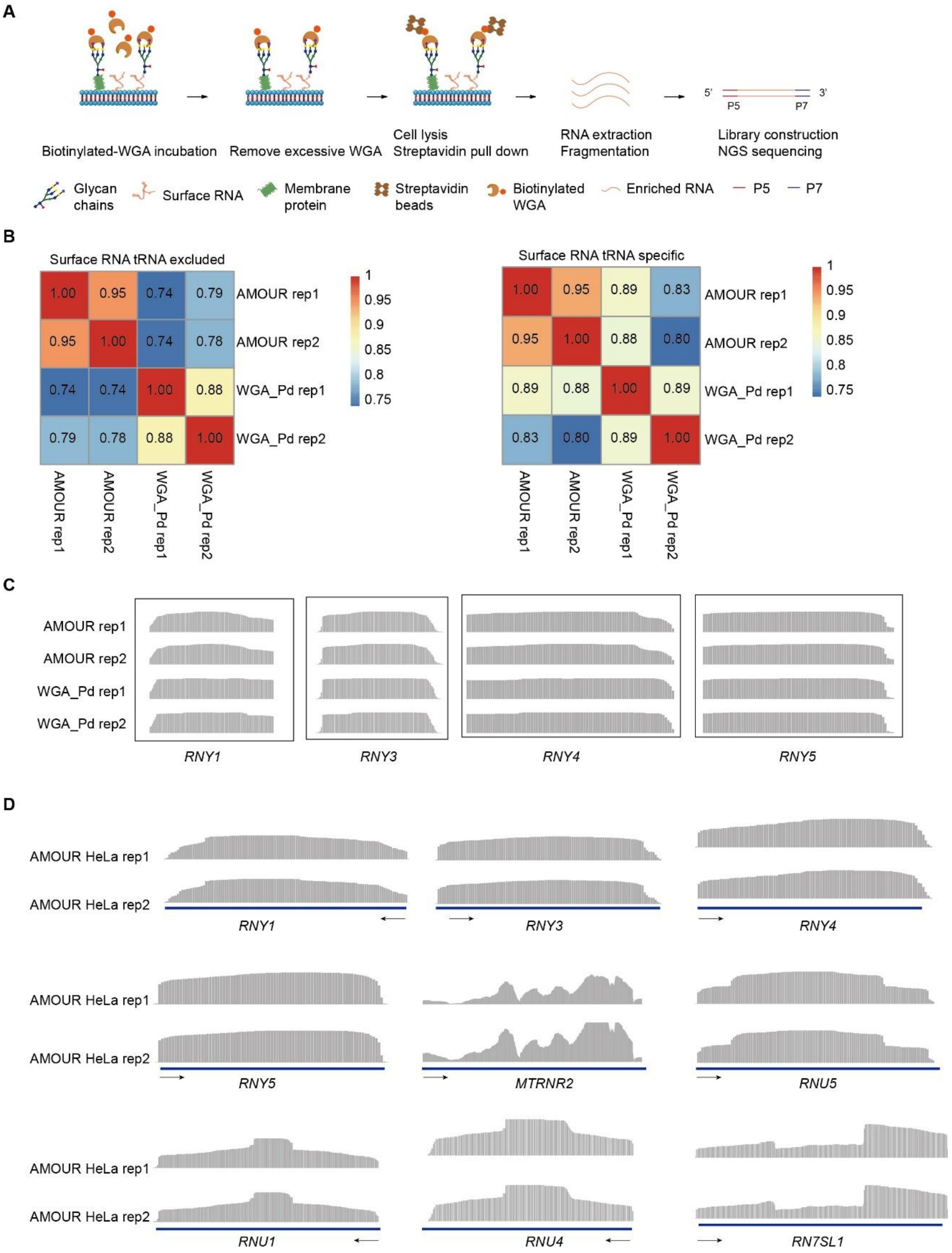

Figure. S3. Validation of ARMOUR via comparative profiling of surface RNAs using biotin-WGA pull down. (A) Schematic flowchart illustrating the Wheat Germ Agglutinin (WGA) assisted membrane-associated RNA pull-down (WGA-Pd) strategy. Biotin-WGA is utilized to enrich membrane-associated RNA, followed by library construction and

sequencing. (B) Heatmap showcasing the correlation among surface RNAs, excluding tRNA (left panel), and tRNA specific (right panel) identified by the AMOUR and WGA-Pd strategies for HEK 293T cells. (C) IGV tracks displaying the reads coverage of representative surface RNAs: *RNY1*, *RNY3*, *RNY4*, and *RNY5* identified by the AMOUR and WGA-Pd strategies for HEK 293T cells. (D) Read coverage patterns for entire genes of several representative surface RNAs identified by AMOUR in HeLa cells, including *RNY1*, *RNY3*, *RNY4*, *RNY5*, *MTRNR2*, *U1*, *U4*, *U5*, and *RN7SL1*. Arrows indicate the direction of transcription.

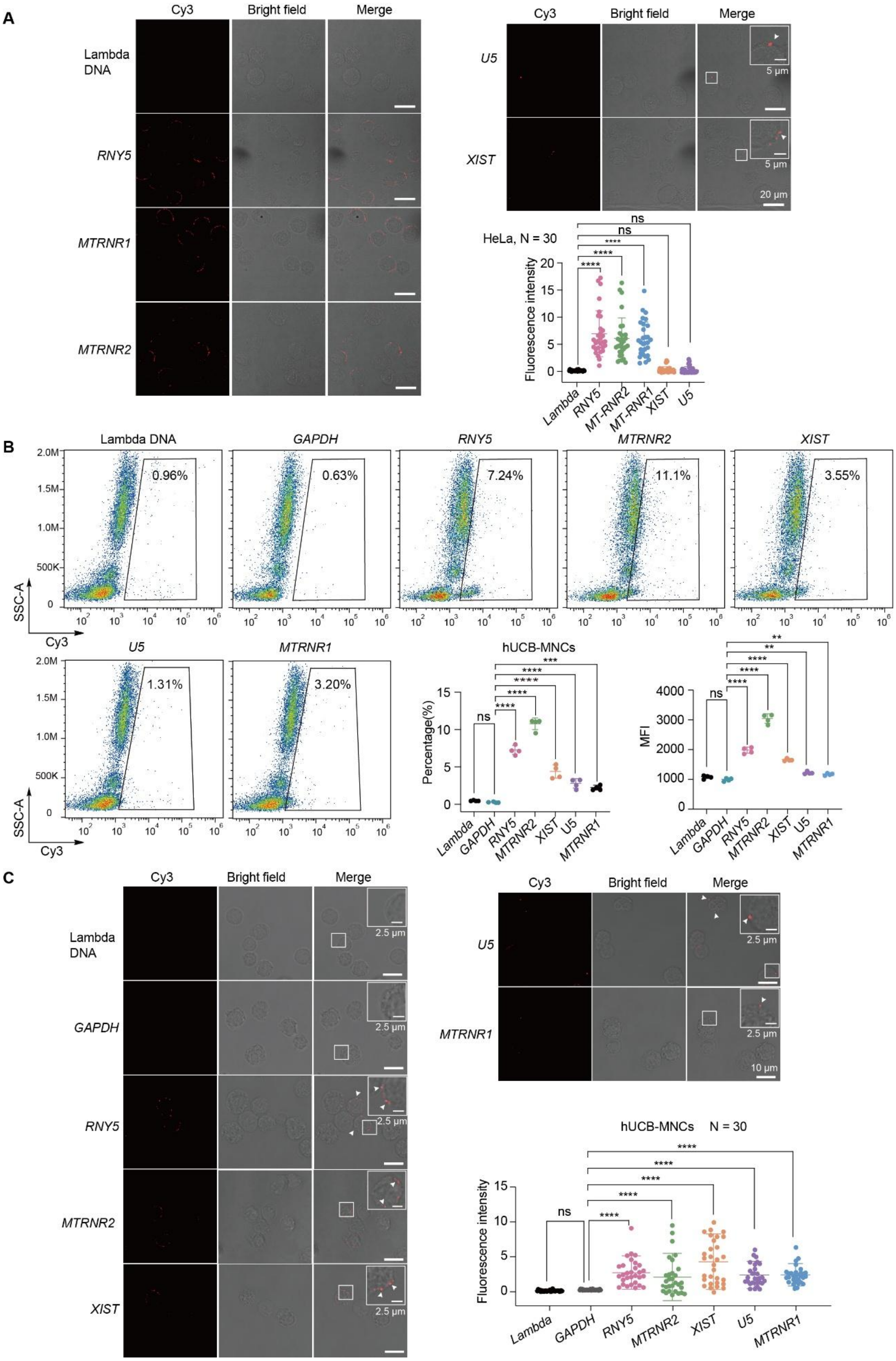
A
Cy3
Bright field
Merge
Lambda DNA
RNY5
MTRNR1
MTRNR2
U5
XIST
5 μm
20 μm
HeLa, N = 30
ns
ns
****
****
****
Fluorescence intensity
0
5
10
15
20
Lambda
RNY5
MT-RNR2
MT-RNR1
XIST
U5
B
Lambda DNA
GAPDH
RNY5
MTRNR2
XIST
0.96%
0.63%
7.24%
11.1%
3.55%
SSC-A
Cy3
2.0M
1.5M
1.0M
500K
0
U5
MTRNR1
1.31%
3.20%
hUCB-MNCs
Percentage(%)
MFI
ns
****
***
**
C
Lambda DNA
GAPDH
RNY5
MTRNR2
XIST
U5
MTRNR1
2.5 μm
10 μm
hUCB-MNCs N = 30
Fluorescence intensity
ns
****
Lambda
GAPDH
RNY5
MTRNR2
XIST
U5
MTRNR1

Figure. S4. Visualization of surface RNA molecules anchored to the outer membrane surface of mammalian cells. (A) Confocal imaging of HeLa cells stained with Cy3-labeled oligonucleotides complementary to specific surface RNAs using Intact-Surface-FISH. White arrowheads denote the precise locations of Intact-Surface-FISH signals. Quantitative analysis of fluorescence intensity for each Cy3-DNA probe labeling is provided. (B) Fluorescence labeling of live hUCB-MNCs with Cy3-Lambda DNA and GAPDH controls, as well as Cy3-DNA probes complementary to RNA *Y5*, *MTRNR2*, *XIST*, *U5*, and *MTRNR1*, using Intact-Surface-FISH. The gated region highlights the cell population exhibiting specific RNAs on the cell surface. Quantitative analysis of cell fraction percentages and mean fluorescence intensity within the Cy3-positive region is provided for each Cy3-DNA probe labeling. (C) Confocal imaging of hUCB-MNCs stained with Cy3-labeled oligonucleotides complementary to representative surface RNAs using Intact-Surface-FISH. White arrowheads indicate the specific locations of Intact-Surface-FISH signals. Quantitative analysis of fluorescence intensity per cell for each Cy3-DNA probe labeling is provided. All flow cytometry analyses represent four independent experiments, and confocal imaging data represent three independent experiments with similar results, shown as mean ± s.d., and analyzed using an unpaired two-tailed Student's t-test; ns: not significant, $**p < 0.01$, $***p < 0.001$, $****p < 0.0001$.

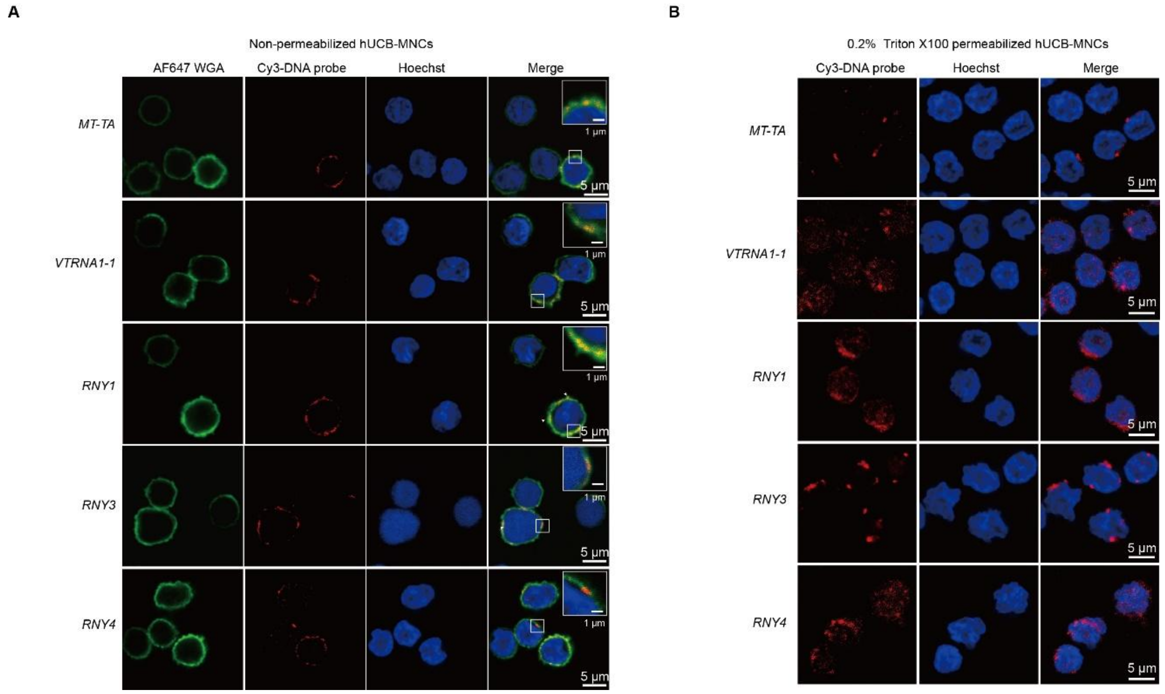

Figure. S5. Validation of representative surface RNA localization. (A) Confocal imaging of live hUCB-MNCs with Cy3-DNA probes complementary to *MT-TA*, *VTRNA1-1*, and *RNAY1/3/4*, using Intact-Surface-FISH. The plasma membrane was labeled with Alexa Fluor 647-labeled WGA. (B) Confocal imaging of fixed and permeabilized hUCB-MNCs using Cy3-DNA probes complementary to representative RNAs was performed with conventional RNA-FISH. All imaging experiments were conducted independently in triplicate, with similar results observed across all repetitions.

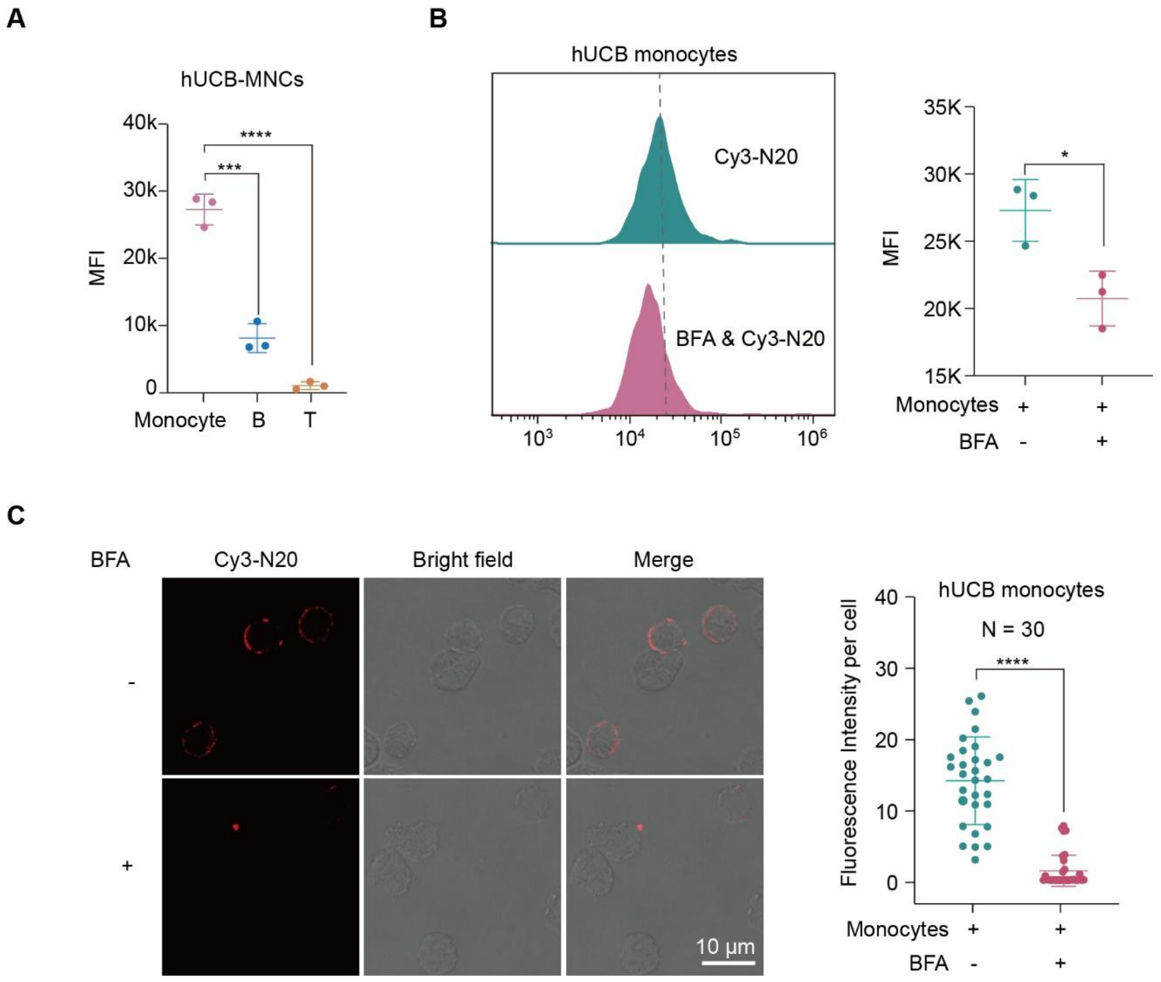

Figure. S6. Analysis of surface RNA localization and transport utilizing live hUCB-MNCs. (A) Quantitative analysis of mean fluorescence intensity (MFI) of live hUCB-MNCs stained with Cy3-Lambda DNA control and Cy3-N20, using Intact-Surface-FISH, followed by antibody staining for the monocyte marker CD14, the B cell marker CD19, and the T cell marker CD3. (B) Fluorescence-activated cell sorting (FACS) analysis of untreated hUCB monocytes and hUCB monocytes pre-treated with Brefeldin A for 6 hours, followed by staining with Cy3-N20 utilizing Intact-Surface-FISH. The left panel shows the FACS results, with the dashed vertical line indicating the Cy3-high population. The right panel provides quantitative analysis of the mean fluorescence intensity within the Cy3-high region for each treatment condition. (C) Imaging of surface RNAs in untreated hUCB monocytes and hUCB monocytes pre-treated with Brefeldin A for 6 hours, followed by staining with Cy3-N20 using Intact-Surface-FISH. Data are presented as the mean ± standard deviation (s.d.) and were analyzed using an unpaired two-tailed Student's t-test; *, $p < 0.05$; ***, $p < 0.001$; ****, $p < 0.0001$.

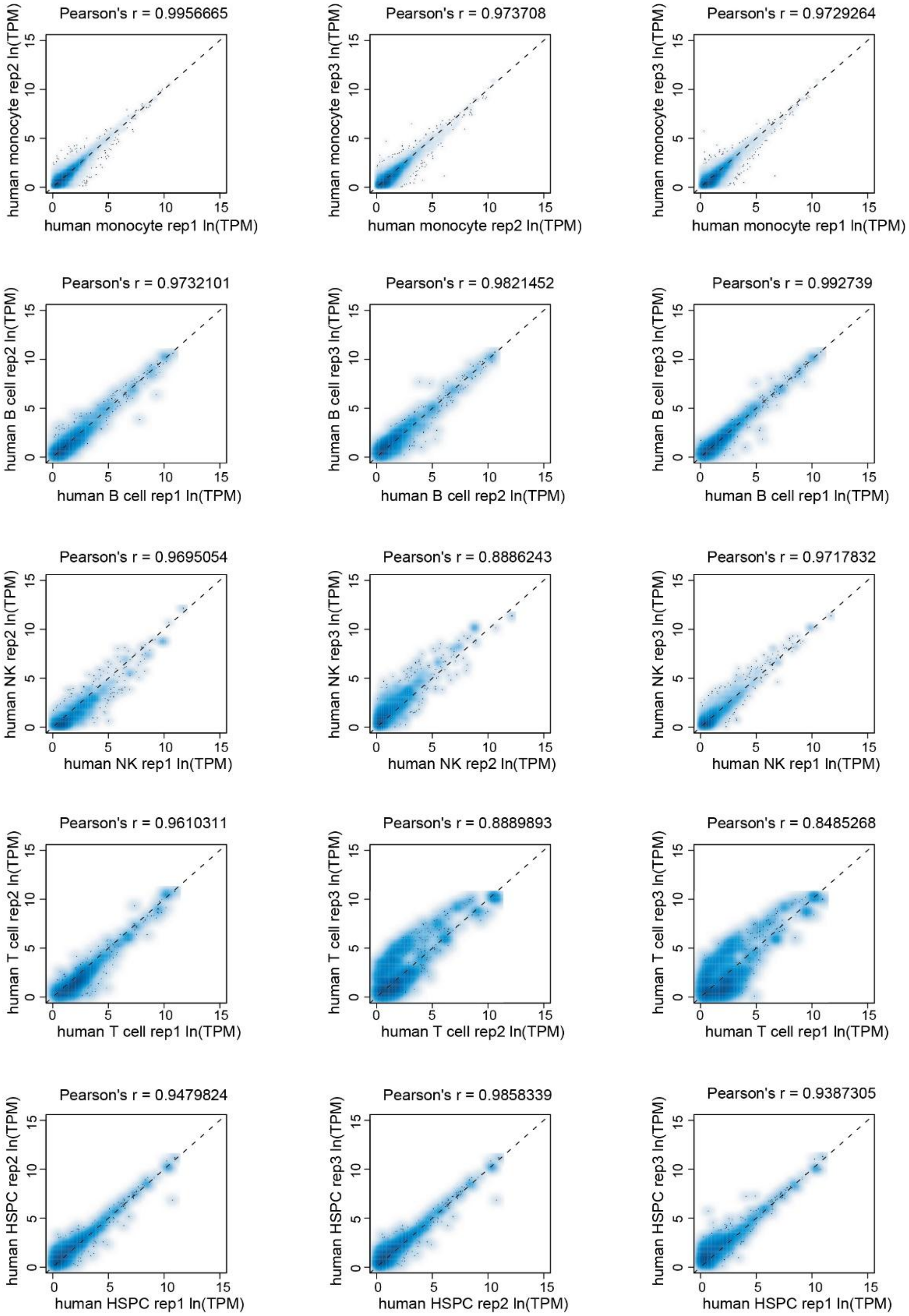

Figure. S7. Pearson correlation analysis of three independent biological replicates of AMOUR datasets across human blood cell types.

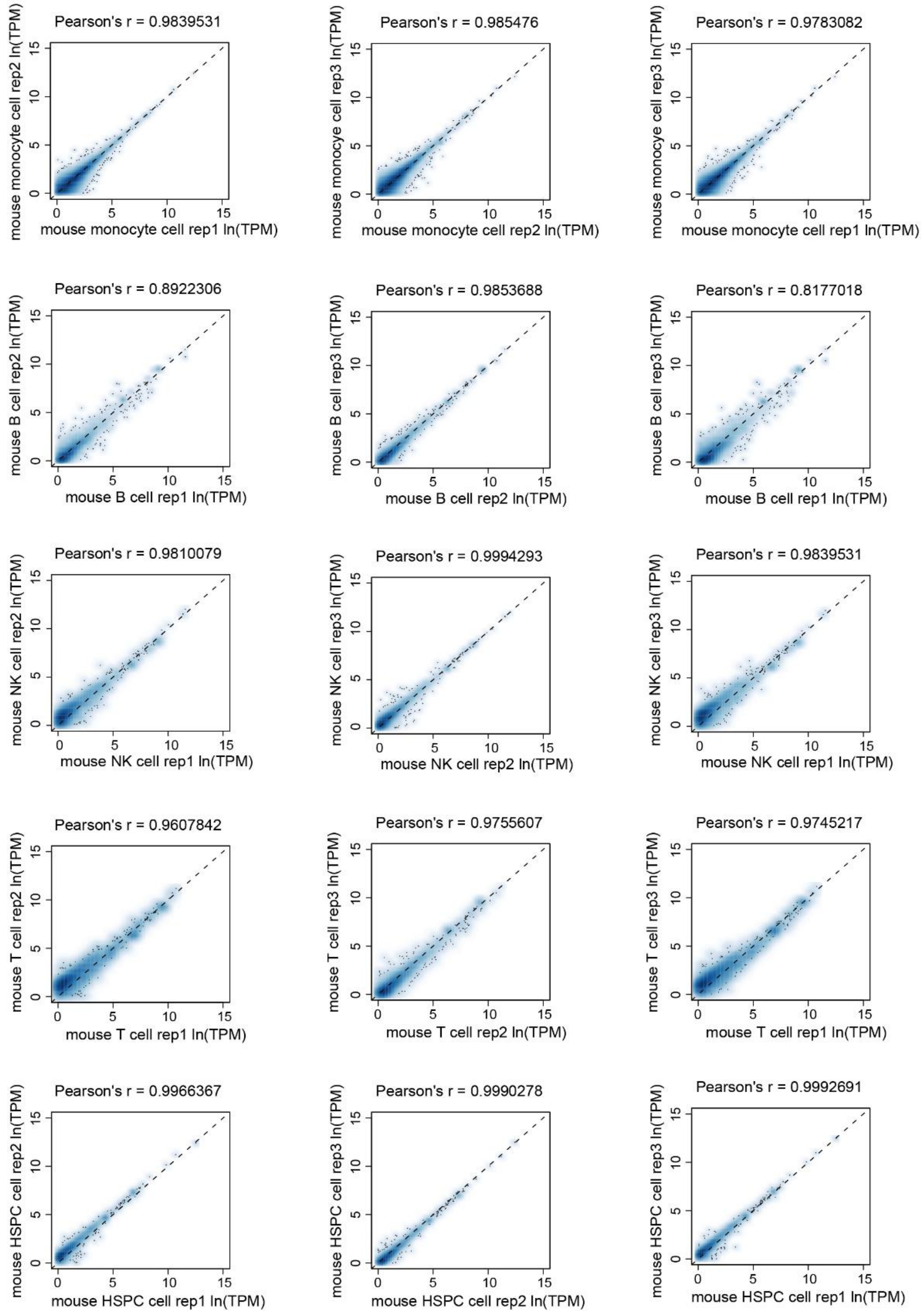

Figure. S8. Pearson correlation analysis of three independent biological replicates of AMOUR datasets across murine blood cell types.

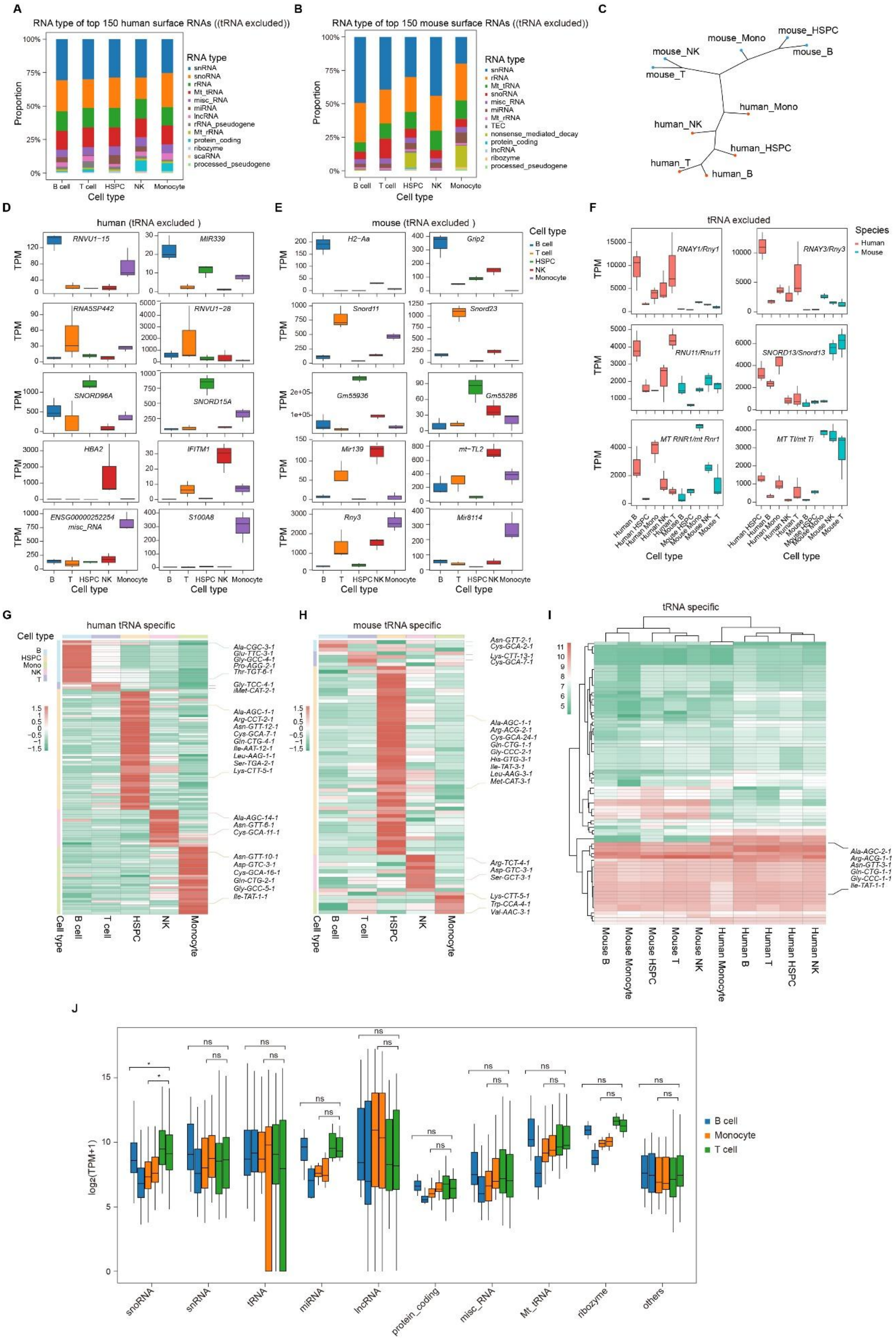
A
RNA type of top 150 human surface RNAs ((tRNA excluded))
RNA type
snRNA
snoRNA
rRNA
Mt_tRNA
misc_RNA
miRNA
lncRNA
rRNA_pseudogene
Mt_rRNA
protein_coding
ribozyme
scaRNA
processed_pseudogene
Proportion
Cell type
B
RNA type of top 150 mouse surface RNAs ((tRNA excluded))
TEC
nonsense_mediated_decay
C
mouse_HSPC
mouse_Mono
mouse_NK
mouse_B
mouse_T
human_Mono
human_NK
human_HSPC
human_T
human_B
D
human (tRNA excluded)
E
mouse (tRNA excluded)
F
tRNA excluded
Species
Human
Mouse
TPM
G
human tRNA specific
H
mouse tRNA specific
I
tRNA specific
J
log2(TPM+1)
B cell
Monocyte
T cell
snoRNA
snRNA
tRNA
miRNA
lncRNA
protein_coding
misc_RNA
Mt_tRNA
ribozyme
others

Figure. S9. Statistics of surface RNAs across diverse blood cell types. (A-B) Stacked plots illustrating the distribution of RNA types among distinct human (A) and murine (B) blood cell subsets, excluding tRNAs. (C) Euclidean distance analysis representing the dissimilarities in surface RNA datasets across distinct blood cell types, excluding tRNAs. (D-E) Box plots presenting distinct highly abundant surface RNA profiles characterizing individual human (D) and murine (E) blood cell populations. (F) Box plot showcasing prevailing highly abundant surface RNA molecules collectively observed across all analysed blood cell types in both human and mouse. (G-I) Heatmap illustrating surface tRNAs identified within distinct human (G) and murine (H) blood cell subsets, along with those conserved across all analysed cell types in both human and mouse (I). Highlighted representative tRNA signatures emphasize discernible expression patterns within these subsets. (J) Box plots depict TPM (transcripts per million) expression values across various RNA categories in B cells, monocytes, and T cells, as identified through total RNA sequencing. Transcripts with an average TPM value greater than 50 were included in the analysis. The number of transcripts in each RNA category is shown at the bottom. Data are presented as the mean ± standard deviation (s.d.) and were analyzed using an unpaired two-tailed Student's t-test; ns, not significant; *: $p < 0.05$.

Document S1. Sequence of the oligonucleotides used in this study.

**Oligonucleotides**	
Primer	Sequence
Oligonucleotides employed in the ARMOUR strategy	
The 325 nt biotin-RNA model	GAAGUUAAAUUAGAUGCUAAAAAUUUGUAAUUAAGA AGGAGGGAUUCGUCAUGUUGGUAUUCCAAAUGCGUAA UGUAGAUAAAACAUCUACUGUUUUGAAACAGACUAAA AACAGUGAUUACGCAGAUAAAUAAAUACGUUAGAUUA AUUCCUACCAGUGACUAAUCUUAUGACUUUUUAAACA GAUAACUAAAAUUACAAACAAAUCGUUUAACUUCUGU AUUUAUUUAUAGAUGUAAUCACUUCAGGAGUGAUUAC AUGAACAAAAAUAUAAAAUAUUCUCAAAACCUUAAGG AUUCACUGGCCGUCGUUUUACAACGUCGUGACUGGGA AAACCCUGGCGUUACCCAACUUAAUCGCCUUGCAGCA CAUCCCCCUUUCGCCAGCUGGCGUAAUAGCGAAGAGG CCCGCACCGAUCGCCCUUCCCAACAGUUGCGCAGCCUG AAUGGCGAAUGGCGCUAACUCGAGGAGCUCCUAGCCC GCCUAAUGAGCGGGCUUUACUAGUCCAUGACCCAGUC ACGUAGCGAUAGCGGAGUGUAUAAUUCUUGAAGACGA AAGGGCCUCGUGAUAC/Biotin/
T7N9 oligo-F	TCACTGTAGTTGTACAGACTCCATAATACGACTCACTAT AGGGTATCAGAGNNNNNNNNN
T7N9 oligo-R	CTCTGATACCCTATAGTGAGTCGTATTATGGAGTCTGTA CAACTACAGTGA
RNA adapter	/5'adenylpyrophosphoryl/AGATCGGAAGAGCGTCGTG/3' Biotin/
cDNA adapter	/5'Phosphorylation/AGATCGGAAGAGCACACGTCTG/3'-Spacer C3/
RT primer	ACACGACGCTCTTCCGATCT
Complementary Oligonucleotides for Intact Surface FISH	

Cy3-20N	Cy3-c30-NNNNNNNNNNNNNNNNNNNN
Cy3-cRNY1-a	Cy3-c30-GAGTAGAACAAGGAGTTCGATCTGTAACTGACTGTG
Cy3-cRNY1-b	Cy3-C30-GCCAGGAGAGTGGAAACTCTCGTAAAAGACTAGTCAAGTG
Cy3-cRNY3-a	Cy3-C30-TGGTTGTGATCAATTAGTTGTAAACACCACTGCACTCGGA
Cy3-cRNY3-b	Cy3-C30-CTAGTCAAGTGAAGCAGTGGGAGTGGAGAAGGAACAAAG
Cy3-cRNY4-a	Cy3-C30-TAATAAGTTCTGATAACCCACTACCATCGGACCAGCCAG
Cy3-cRNY4-b	Cy3-C30-CAAATGGTAAAAAGCCAGTCAAATTTAGCAGTGGG
Cy3-cRNY5-a	Cy3-C30-ACACTCGGACCAACTGTGTTATCCTGTGAGTCTCTTGTTA
Cy3-cRNY5-b	Cy3-C30-TGTTATTAGTGCAAAACAGCAAGCTAGTCAAGCGCGGTTG
Cy3-cMN-RNR1-a	Cy3-C30-GTGTGGCTAGGCTAAGCGTTTTGAGCTGCATTGCT
Cy3-cMTRNR1-b	Cy3-C30-TTGAGTTTTAAGCTGTGGCTCGTAGTGTTCTGGCGAGCA
Cy3-cMNcRNR2-a	Cy3-C30-CTCTTTTAGCTGTTCTTAGGTAGCTCGTCTGGTTTCG
Cy3-cMNcRNR2-b	Cy3-C30-TGTTGAGCTTGAACGCTTTCTTAATTGGTGGCTGCT
Cy3-cXISTexon1-a	Cy3-C30-CTTATCGTAGTGGCCAGAGTGGTAGAAGAGATACGGAG
Cy3-cXISTexon1-b	Cy3-C30-CAGGAGTAGCGTTGGCACAGTCCACCAAATTATTTG
Cy3-cXISTexon2	Cy3-C30-ATCTTCCTATCTGGGACCAGGAAAGTATCTTGACAGAACC
Cy3-cXIST-exon3-a	Cy3-C30-CCTGGGTCTGACTTGCCTTCAGTATTTTGCACAGT
Cy3-cXIST-exon3-b	Cy3-C30-TATGCCTGTGGTCACTTACAACTGTGCACCTTGATTGTCC
ATTO590-cRNY1-a	ATTO590-c30-GAGTAGAACAAGGAGTTCGATCTGTAACTGACTGTG
ATTO590-cRNY1-b	ATTO590-C30-GCCAGGAGAGTGGAAACTCTCGTAAAAGACTAGTCAAGTG
ATTO590-cRNY3-a	ATTO590-C30-TGGTTGTGATCAATTAGTTGTAAACACCACTGCACTCGGA
ATTO590-cRNY3-b	ATTO590-C30-CTAGTCAAGTGAAGCAGTGGGAGTGGAGAAGGAACAAAG

ATTO590-cRNY4-a	ATTO590-C30-TAATAAGTTCTGATAACCCACTACCATCGGACCAGCCAG
ATTO590-cRNY4-b	ATTO590-C30-CAAATGGTAAAAAGCCAGTCAAATTTAGCAGTGGG
ATTO590-cRNY5-a	ATTO590-C30-ACACTCGGACCAACTGTGTTATCCTGTGAGTCTCTTGTTA
ATTO590-cRNY5-b	ATTO590-C30-TGTTATTAGTGCAAAACAGCAAGCTAGTCAAGCGCGGTTG
ATTO590-cMN-RNR1-a	ATTO590-C30-GTGTGGCTAGGCTAAGCGTTTTGAGCTGCATTGCT
ATTO590-cMTRNR1-b	ATTO590-C30-TTGAGTTTTAAGCTGTGGCTCGTAGTGTTCTGGCGAGCA
ATTO590-cMNcRNR2-a	ATTO590-C30-CTCTTTTAGCTGTTCTTAGGTAGCTCGTCTGGTTTCG
ATTO590-cMNcRNR2-b	ATTO590-C30-TGTTGAGCTTGAACGCTTTCTTAATTGGTGGCTGCT
ATTO590-cXISTexon1-a	ATTO590-C30-CTTATCGTAGTGGCCAGAGTGGTAGAAGAGATACGGAG
ATTO590-cXISTexon1-b	ATTO590-C30-CAGGAGTAGCGTTGGCACAGTCCACCAAATTATTTG
ATTO590-cXISTexon2	ATTO590-C30-ATCTTCCTATCTGGGACCAGGAAAGTATCTTGACAGAACC
ATTO590-cXIST-exon3-a	ATTO590-C30-CCTGGGTCTGACTTGCCTTCAGTATTTTGCACAGT
ATTO590-cXIST-exon3-b	ATTO590-C30-TATGCCTGTGGTCACTTACAACTGTGCACCTTGATTGTCC

Table S1 (separate file). Summary of surface RNA characteristics in HeLa and HEK 293T cells.

Table S2 (separate file). Summary of surface RNA profiles across human blood cell types.

Table S3 (separate file). Summary of surface RNA profiles across murine blood cell types, excluding tRNA.

Table S4 (separate file). Summary of cell type-specific surface RNAs in human and murine blood cells, excluding tRNA.

Table S5 (separate file). Summary of cell type-specific surface tRNAs in human and mouse blood cells.

Video S1 (separate file). 3D imaging of surface RNA *MTRNR2* (green) and plasma membrane tracker WGA (red) in hUCB-MNCs with Intact-Surface-FISH, utilizing STED microscopy STEDYCON.

Video S2 (separate file). 3D imaging of surface RNA *XIST* (green) and plasma membrane tracker WGA (red) in hUCB-MNCs with Intact-Surface-FISH, utilizing STED microscopy STEDYCON.

Video S3 (separate file). 3D imaging of surface RNA *RNY5* (green) and plasma membrane tracker WGA (red) in hUCB-MNCs with Intact-Surface-FISH, utilizing STED microscopy STEDYCON.

References

Bolger, A.M., Lohse, M., and Usadel, B. (2014). Trimmomatic: a flexible trimmer for Illumina sequence data. Bioinformatics 30, 2114-2120.
Bray, N.L., Pimentel, H., Melsted, P., and Pachter, L. (2016). Near-optimal probabilistic RNA-seq quantification. Nat Biotechnol 34, 525-527.
Dobin, A., Davis, C.A., Schlesinger, F., Drenkow, J., Zaleski, C., Jha, S., Batut, P., Chaisson, M., and Gingeras, T.R. (2013). STAR: ultrafast universal RNA-seq aligner. Bioinformatics 29, 15-21.
Ge, R., Ye, C., Peng, Y., Dai, Q., Zhao, Y., Liu, S., Wang, P., Hu, L., and He, C. (2023). m^6A-SAC-seq for quantitative whole transcriptome m^6A profiling. Nat Protoc 18, 626-657.
Hu, L.L., Liu, S., Peng, Y., Ge, R.Q., Su, R., Senevirathne, C., Harada, B.T., Dai, Q., Wei, J.B., Zhang, L.S., *et al.* (2022). m^6A RNA modifications are measured at single-base resolution across the mammalian transcriptome (vol 40, pg 1210, 2022). Nature Biotechnology.
Huang, N., Fan, X., Zaleta-Rivera, K., Nguyen, T.C., Zhou, J., Luo, Y., Gao, J., Fang, R.H., Yan, Z., Chen, Z.B., *et al.* (2020). Natural display of nuclear-encoded RNA on the cell surface and its impact on cell interaction. Genome Biol 21, 225.
Love, M.I., Huber, W., and Anders, S. (2014). Moderated estimation of fold change and dispersion for RNA-seq data with DESeq2. Genome Biol 15, 550.
Pelts, M., Pandya, S.M., Oh, C.J., and Model, M.A. (2011). Thickness profiling of formaldehyde-fixed cells by transmission-through-dye microscopy. Biotechniques 50, 389-396.
Wu, T., Hu, E., Xu, S., Chen, M., Guo, P., Dai, Z., Feng, T., Zhou, L., Tang, W., Zhan, L., *et al.* (2021). clusterProfiler 4.0: A universal enrichment tool for interpreting omics data. Innovation (Camb) 2, 100141.
Zhou, Y., Zhou, B., Pache, L., Chang, M., Khodabakhshi, A.H., Tanaseichuk, O., Benner, C., and Chanda, S.K. (2019). Metascape provides a biologist-oriented resource for the analysis of systems-level datasets. Nat Commun 10, 1523.
